# Identification of a Force‐Induced Sox9^+^Acan^+^ Transitional Subpopulation Linked to FGF2–FGFR2–ERK Signaling in Orthodontic Bone Remodeling

**DOI:** 10.1002/advs.202519330

**Published:** 2026-01-22

**Authors:** Miao Tan, Minyu He, Mingrui Zong, Qiya Tang, Yinan Liu, Jiaju Deng, Shun Huang, Xiaoxiao Lei, Jie Li, Lan Huang

**Affiliations:** ^1^ College of Stomatology Chongqing Medical University Chongqing P. R. China; ^2^ Chongqing Key Laboratory of Oral Diseases Chongqing Medical University Chongqing P. R. China; ^3^ Chongqing Municipal Key Laboratory of Oral Biomedical Engineering of Higher Education Chongqing Medical University Chongqing P. R. China; ^4^ Chongqing Municipal Health Commission Key Laboratory of Oral Biomedical Engineering Chongqing Medical University Chongqing P. R. China

**Keywords:** crosstalk, FGF signaling, mesenchymal lineage cell, orthodontic tooth movement, single‐cell RNA sequencing

## Abstract

Orthodontic tooth movement (OTM) under excessive force is often accompanied by orthodontically induced inflammatory root resorption (OIIRR). Multiple cell types and pathways contribute, yet the heterogeneity of mesenchymal lineage cells remains poorly defined. Murine models of OTM are established, and single‐cell RNA sequencing (scRNA‐seq) is performed to profile force‐induced transcriptional dynamics. Mesenchymal lineage cells are resolved into five subsets, including mesenchymal stem cells, cementoblasts, osteoblasts, fibroblasts, and a previously unrecognized cluster co‐expressing Sox9 and Acan. Functional validation by RNAscope, multiplex immunohistochemistry, and mechanostimulation confirms the localization and activity of this subpopulation. scRNA‐seq also identifies 14 additional cell types, including immune and mesenchymal populations. Reclustering of macrophages reveals gene programs associated with bone resorption. Sox9^+^Acan^+^ cells exemplify a mechanosensitive transitional population that integrates biomechanical stress with osteoimmune regulation, a paradigm relevant to skeletal mechanobiology. These cells interact with Mmp14^+^ macrophages to activate FGF2–FGFR2–ERK signaling, thereby enhancing osteoclast differentiation and bone resorption. A GelMA@siRNA hydrogel system for localized delivery of Sox9‐targeting siRNA silences *Sox9* expression in vivo, suppresses osteoclast activity, reduces root resorption, and modulates tooth movement. Together, these findings identify Sox9^+^Acan^+^ cells as a force‐sensitive regulatory node in skeletal biology and propose a therapeutic strategy to mitigate OIIRR.

## Graphical abstract

Mechanical loading induces a previously unrecognized Sox9^+^Acan^+^ transitional mesenchymal cell population in the periodontal ligament that promotes osteoclastogenesis via the FGF2–FGFR2–ERK axis. Targeting this mechanoresponsive stromal population using a localized GelMA@siRNA delivery strategy attenuates pathological osteoclast overactivation and root resorption while preserving effective tooth movement. This study reveals a mechanobiological framework linking mesenchymal plasticity to osteoimmune regulation during orthodontic bone remodeling.

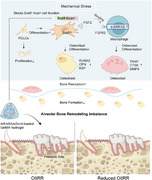

## 1 Introduction

Orthodontic tooth movement (OTM) is a mechanically driven biological process dependent on coordinated bone remodeling within the periodontal microenvironment, involving resorptive activity on the compression side and osteogenic activity on the tension side [1, 2]. Clinically, the application of controlled force to achieve tooth movement is frequently accompanied by varying degrees of orthodontically induced inflammatory root resorption (OIIRR) [3]. Excessive or poorly controlled forces amplify inflammatory responses and tissue damage, accelerating root resorption and jeopardizing long‐term periodontal stability [2]. Risk and severity rise with fixed appliances, under heavy force, intrusion, torque, extensive treatment duration, extraction space closure, or large apical displacement, especially of maxillary incisors [4]. It is now understood that inflammatory mediators detected in resorbed roots, the periodontal ligament (PDL), and alveolar bone under heavy force are central to OIIRR pathogenesis [1]. Numerous cell types, including periodontal ligament cells (PDLCs), osteoblasts, cementoblasts, and immune cells, are involved in this process. Yet the specific cellular heterogeneity of the periodontal microenvironment that demarcates physiological remodeling from pathological root resorption remains unclear.

Recent research has focused primarily on immune cell contributions to OTM, describing how immune heterogeneity influences remodeling [5]. It has been established that Ccr2^+^ macrophages promote OTM and alveolar bone remodeling, with the CCL signaling pathway playing a crucial role [6]. A recent report revealed C3ar1^+^ macrophage subclusters enriched for positive regulation of the mitogen‐activated protein kinase (MAPK) cascade, which aligns with augmented osteoclast activity at pressure sites [7]. Additional studies show that γδT cells can increase IL‐17A, recruit monocytes and neutrophils, thereby expanding osteoclast populations [8]. Separately, extracellular vesicles from M2 macrophages have been reported to reprogram neutrophils toward an Anxa1^hi^ state, which supports inflammatory bone regeneration [9]. These immune‐centered insights clarify key mechanisms of force translation and highlight the extensive influence of inflammatory control over bone dynamics. Yet immune programs operate within the PDL and alveolar bone, where mesenchymal lineage cells shape the local environment [10]. The contribution of these mesenchymal cells to periodontal remodeling remains comparatively underexplored.

Within this context, mesenchymal lineage cells are prime orchestrators of orthodontic bone remodeling and immune calibration, given their defining attributes of pluripotency, heterogeneity, and mechanosensitivity. Emerging evidence from lineage tracing and reporter models has identified Gli1^+^ and Axin2^+^ progenitors in force‐responsive periodontal and alveolar niches, linked their recruitment and fate decisions to spatial gradients of resorption and formation, and revealed contributions of undifferentiated cells around the neurovascular bundle to alveolar bone, cementum, and the periodontal ligament [11, 12, 13, 14, 15, 16]. Functional experiments further demonstrated mesenchymal control of the inflammatory milieu and osteoclastogenesis through prostaglandin E_2_ induction of macrophage interleukin 10 and through exosomal microRNA‐1246 acting via Nfat5 to restore the balance between Th17 and Treg cells, with additional bidirectional crosstalk supporting resolution and homeostasis [17, 18, 19, 20, 21]. Single‐cell analyses complement these findings by resolving mesenchymal diversity and transient states in oral and periodontal tissues [22, 23, 24, 25, 26, 27], For instance, progenitor subsets such as PTHrP^+^ cells, identified in the early developmental stages and shown not to interconvert with other cell types, secrete PTHrP in an autocrine or paracrine manner, and thereby influencing a range of periodontal cell populations. Overall, these findings collectively suggest that mesenchymal subset composition and state transitions are fundamental regulators of the balance between osteoblasts and osteoclasts, with implications that extend beyond the periodontium to bone biology more broadly. This underscores the utility of scRNA‐seq in defining subset‐specific molecular programs and mechanosensitive communication pathways channels under orthodontic force.

Guided by these considerations, we applied scRNA‐seq to periodontal tissues subjected to orthodontic force and constructed an integrated mesenchymal and immune cell atlas. The analysis resolved mesenchymal heterogeneity in the force‐responsive niche and identified a previously unrecognized mechanosensitive transitional subpopulation that co‐expresses *SRY‐box transcription factor 9* (*Sox9*) and *Aggrecan* (*Acan*). Beyond the periodontal context, this population exemplifies a broader principle of force‐responsive stromal regulation, demonstrating how skeletal tissues harness specialized mesenchymal intermediates to couple mechanical stress with osteoimmune dynamics. The differentiation trajectories of these Sox9^+^Acan^+^ cells were delineated, along with their communication with Mmp14^+^ macrophages, a population known to drive bone remodeling and root resorption. Functional validation showed that this subpopulation promotes remodeling through the fibroblast growth factor 2 (FGF2)–fibroblast growth factor receptor 2 (FGFR2)–extracellular signal‐regulated kinase (ERK) pathway. To further explore translational potential, we developed a methoxy‐poly (ethylene glycol)‐modified human serum albumin/small interfering RNA against *Sox9* (m5‐hSA/si*Sox9*)‐loaded gelatin methacryloyl (GelMA) sustained‐release system (GelMA@siRNA) to selectively modulate *Sox9* expression or target this subpopulation, with the aim of mitigating root resorption. By positioning Sox9^+^Acan^+^ cells as a model of force‐sensitive mesenchymal regulation, this work contributes to a generalizable framework in bone biology while also supplying a therapeutic strategy for OIIRR.

## 2 Results

### 2.1 scRNA‐seq Reveals Mechanosensitive Sox9^+^Acan^+^ Cells

**FIGURE 1:**
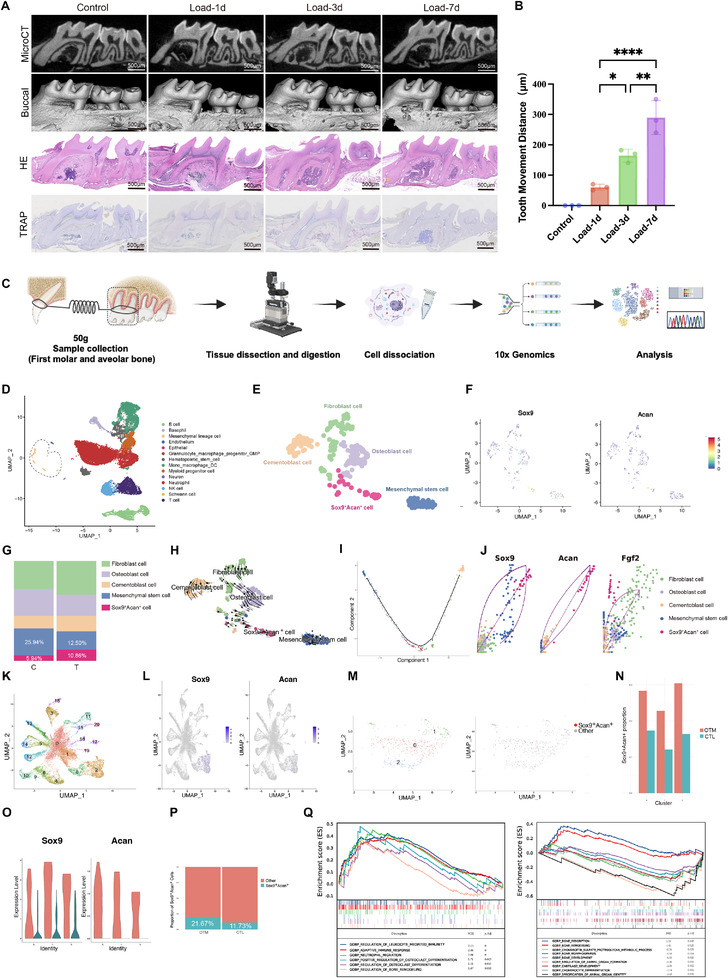
Establishment of a high‐force OTM model and scRNA‐seq analysis of periodontal tissues. (A–B) Representative Micro‐CT, buccal view, H&E, and TRAP staining of maxillary first molars under control and force loading for 1, 3, and 7 days, together with the quantification of tooth movement distance (mean ± SD; n = 3–6 mice per group; one‐way ANOVA with Tukey's post‐hoc test. ^*^ *p* < 0.05, ^**^ *p* < 0.01, ^***^ *p* < 0.001, ^****^ *p* < 0.0001). Yellow arrows in the H&E images indicate the sites of root resorption. (C) Workflow of scRNA‐seq, including tissue dissection, enzymatic digestion, 10× Genomics library preparation, and downstream analysis. Periodontal tissues from 10 mice were pooled for each group (Control and Load‐1d) to generate single‐cell suspensions. (D–F) UMAP plots showing identified clusters and representative marker gene expression. (G) Stacked bar plot showing the proportional distribution of mesenchymal lineage subtypes in control (C) and loaded (T) groups. (H–I) Pseudotime trajectory and RNA velocity analysis delineate the differentiation dynamics of the *Sox9^+^Acan^+^* population. (J) Expression patterns of *Sox9*, *Acan*, and *Fgf2* plotted along the inferred transcriptional trajectory, with cells colored by mesenchymal subtypes. (K–P) Independent reanalysis of the publicly available dataset GSE287729, which contains alveolar bone tissues from both control (non‐loading) and orthodontic loading 7 d groups, revealed enrichment of Sox9 and Acan expression in specific subclusters. (K–L) UMAP visualization of identified clusters and Sox9, Acan marker gene expression. (M–N) UMAP showing reclustering results for Cluster 2 and the distribution of *Sox9^+^Acan^+^* double‐positive cells. (O) Violin plots showing Sox9 and Acan expression across all subclusters. (P) Proportion of Sox9^+^Acan^+^ double‐positive cells within Cluster 2 in the control and loading groups, respectively. (Q) GSEA reveals enriched biological processes and pathways. For scRNA‐seq–based analyses (C–Q), data are presented descriptively; no statistical testing was applied unless explicitly stated.

OTM models under excessive force (50 g) were established across multiple time points. Model validation by Micro‐CT, Hematoxylin and Eosin (H&E) staining, and TRAP staining confirmed progressive root resorption and a time‐dependent increase in osteoclast activity (Figure 1A), paralleled by steadily advancing tooth displacement (Figure 1B). To characterize early cellular responses, tissues harvested at 1 d post‐loading were defined as the treatment (T) group, and non‐loaded samples served as controls (C). Gingival tissue was removed from the first molars. The first molars, periodontal ligament, and approximately 1 mm of surrounding buccal and palatal alveolar bone were subjected to scRNA‐seq (Figure 1C). After quality control, 24,227 cells were obtained (T, n = 14,168 cells; C, n = 10,059 cells). The uniform manifold approximation and projection (UMAP) method was applied to visualize data. A total of 14 cell lineages were identified, including macrophages, mesenchymal lineage cells, endothelial cells, hematopoietic stem/progenitor cells, and Schwann cells, etc. (Figure 1D). Marker genes of mesenchymal lineage cells and macrophages are shown in Figure S1.

Subclustering of mesenchymal lineage cells revealed five subsets, which included osteoblasts, cementoblasts, mesenchymal stem cells (MSCs), fibroblasts, and a previously unreported Sox9^+^Acan^+^ subpopulation (Figure 1E,F). In the T group, Sox9^+^Acan^+^ cells accounted for 10.86% of mesenchymal lineage cells, compared to 5.94% in the C group, representing an approximate twofold increase. Concurrently, mesenchymal stem cells accounted for 25.94% in the C group but dropped to 12.5% in the T group, representing nearly a 50% reduction (Figure 1G). RNA velocity analysis further supported this interpretation by showing a clear directional flow from MSCs toward the Sox9^+^Acan^+^ state, indicating active induction of this transitional program (Figure 1H). Downstream dynamics from the Sox9^+^Acan^+^ population toward osteoblast‐ and fibroblast‐like clusters were detectable but modest, consistent with a transient, stress‐responsive phenotype rather than a committed lineage. Also, pseudotime analysis positioned MSCs at the trajectory origin with high stemness, cementoblasts at the endpoint, and Sox9^+^Acan^+^ cells intermediately, suggesting a transitional state (Figure 1I). Along the inferred transcriptional trajectory, *Sox9* and *Acan* exhibited a coordinated increase, reaching peak expression within the Sox9^+^Acan^+^ transitional population. *Fgf2* expression also rose sharply within the Sox9^+^Acan^+^ population, hinting at a potential signaling involvement associated with this state. The functional relevance of this pattern is further explored in subsequent sections (Figure 1J).

To assess cross‐dataset reproducibility, we reanalyzed the publicly available scRNA‐seq dataset GSE287729 (single‐cell transcriptomes of mouse periodontal tissues subjected to 7 days of orthodontic force compared with untreated controls). Importantly, dimensionality reduction and clustering identified 20 distinct cell clusters (Figure 1K). Sox9 and Acan expression was predominantly enriched in Cluster 2 (Figure 1L), which was further confirmed in the supplementary analysis (Figure S2E,F). Further subclustering of Cluster 2 resolved three subpopulations (Figure 1M). The number of Sox9^+^Acan^+^ cells doubled, and their average expression level was also increased in the OTM group (Figure 1N–P).

This pattern was mirrored in human data. Analysis of the SHED scRNA‐seq dataset (GSE221216) revealed that Sox9^+^Acan^+^ double‐positive cells represent a recurrent, though minority, transcriptional state. This state was distributed across multiple mesenchymal clusters, with peak enrichment observed in Cluster 4 (∼8.6%). This finding indicates that the Sox9^+^Acan^+^ state is selectively enriched in specific human SHED‐derived mesenchymal subsets rather than restricted to a fixed lineage (Figure S2H–K). Besides, re‐analysis of a public mouse periodontal ligament scRNA‐seq dataset (GSE160358 and GSE168450) confirmed a congruent cluster co‐expressing Sox9 and Acan (Figure S2A–D). Functional analysis of the Sox9^+^Acan^+^ population in our scRNA‐seq dataset via GSEA revealed its association with bone resorption and remodeling pathways under force, concurrent with suppressed chondrogenesis (Figure 1Q), underscoring its principal role in periodontal adaptation during OTM. These findings collectively identify the Sox9^+^Acan^+^ population as a key mechanosensitive transitional cell type that coordinates bone remodeling and immune modulation during orthodontic tooth movement.

### 2.2 Force Increases Sox9^+^Acan^+^ Cells Promoting Osteoclastogenesis and Immunomodulation

**FIGURE 2:**
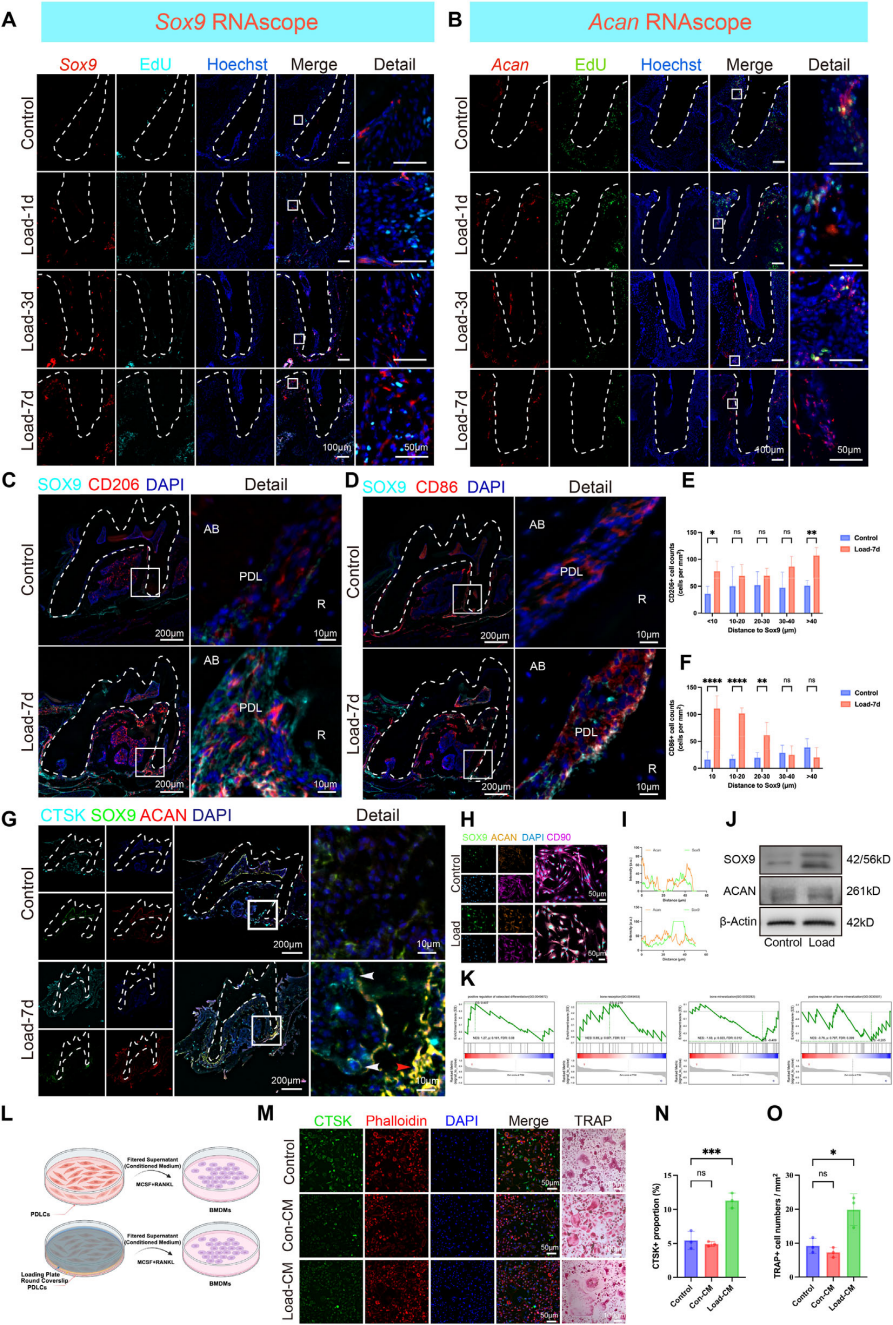
Sox9^+^Acan^+^ cells are activated by orthodontic force in vivo. (A–B) RNAscope in situ hybridization for *Sox9* and *Acan* with EdU at the indicated loading times. (C–D) Immunofluorescence of SOX9 with macrophage markers CD206 (M2) and CD86 (M1). (E–F) Representative images and quantification of the spatial proximity between macrophages and Sox9^+^Acan^+^ cells. (G) Multiplex IHC staining of CTSK, SOX9, and ACAN in vivo. Red arrows indicate Sox9^+^Acan^+^ cells, and white arrows indicate CTSK^+^ cells. (H–J) Immunofluorescence of SOX9, ACAN, and CD90 in PDLCs and Western blot validation of SOX9 and ACAN under loading. (K) GSEA enrichment plots for individual osteogenic‐ and osteoclast‐associated pathways (e.g., GO:0045672 and GO:0030282), calculated for the Sox9^+^Acan^+^ subpopulation. (L) Schematic of the conditioned‐medium (CM)‐induced osteoclastogenesis assay. (M) Representative images of CM‐induced osteoclastogenesis. (N–O) Quantification of CTSK^+^ cells and TRAP^+^ osteoclasts in vitro. Data presentation and n: A–G (in vivo), mean ± SD; n = 3–6 mice per group. H–O (in vitro), n = 3 independent biological experiments; data are shown as mean ± SD. Statistics: For all quantitative comparisons shown in this figure (except panels K and L), unpaired two‐tailed Student's t‐test (two groups) or one‐way ANOVA with Tukey's post‐hoc test (multiple groups) were used. Panels K and L are descriptive and were not subjected to statistical testing. Significance notation: ns, not significant (*P* ≥ 0.05); ^*^ *p* < 0.05; ^**^ *p* < 0.01; ^***^ *p* < 0.001; ^****^ *p* < 0.0001.

To evaluate the spatial distribution and proliferation of Sox9^+^Acan^+^ cells in vivo, RNAscope and 5‐ethynyl‐2′‐deoxyuridine (EdU) double labeling were performed. The results showed that these cells were predominantly located on the pressure side of the PDL, and their abundance increased with the duration of force application (Figure 2A,B). A multiplex RNAscope assay further confirmed the pressure‐side localization of Sox9^+^Acan^+^ cells, particularly near the distal root and apex of the first molar, and demonstrated a marked increase in double‐positive cells in the 7‐day force group compared to controls (Figure S3A). However, no corresponding increase in Sox9^+^Acan^+^EdU^+^ cells was observed over time (Figure S3B), confirming that their population expansion was not due to proliferation. Spatial analysis of macrophage infiltration showed a significantly increased presence of CD86^+^ (M1) macrophages near Sox9^+^ cells, with no notable change in CD206^+^ (M2) macrophages (Figure 2C–F). Furthermore, multiplex immunohistochemistry (mIHC) revealed an elevated M1/M2 ratio after 7 days of force application (Figure S3C). Besides, mIHC for SOX9, ACAN, and cathepsin K (CTSK) revealed a distinct spatial pattern in the pressure zone (Figure S3D). After 7 days of force application, root resorption was observed. In addition to CTSK^+^ osteoclasts, SOX9^+^ACAN^+^ double‐positive cells were also detected within the resorption lacunae (Figure 2G). This spatial adjacency suggests a potential paracrine mode of communication. A similar spatial relationship was observed in a separate triple staining for SOX9, ACAN, and osteopontin (OPN) (Figure S3E).

To investigate the direct effects of mechanical force on PDLCs, mouse PDLCs were isolated and their identity confirmed through flow cytometry and colony‐forming assays (Figure S4A–H). Application of 2 g/cm^2^ static pressure for 24 h induced co‐localization of SOX9 and ACAN, along with an increase in double‐positive cells (Figure 2H,I). Western blot analysis confirmed elevated SOX9 protein expression under mechanical loading (Figure 2J). Osteogenic induction assays demonstrated reduced mineralization capacity in mechanical force‐treated mouse PDLCs, as evidenced by alkaline phosphatase (ALP) and Alizarin Red S (ARS) staining (Figure S4K,L). GSEA of this cell subset revealed significant enrichment and upregulation of osteoclast differentiation pathways (Figure 2K). To examine whether mechanical force‐activated mouse PDLCs influence osteoclastogenesis via paracrine signaling, conditioned medium (CM) was collected from mechanically stimulated mouse PDLCs and applied to bone marrow‐derived macrophages (BMDMs) during osteoclast differentiation (Figure 2L). Phalloidin and CTSK double staining, tartrate‐resistant acid phosphatase (TRAP) staining, and immunofluorescence‐based (IF) quantification confirmed that the conditioned medium significantly enhanced osteoclast formation (Figure 2M–O). Overall, these results indicate that mechanical force induces a non‐proliferative expansion of Sox9^+^Acan^+^ cells in the periodontal ligament, and that this population promotes osteoclastogenesis via paracrine signaling and alters the spatial distribution of macrophages.

### 2.3 Sox9 Manipulation Alters Mouse PDLCs Osteogenesis and Paracrine Signaling

**FIGURE 3:**
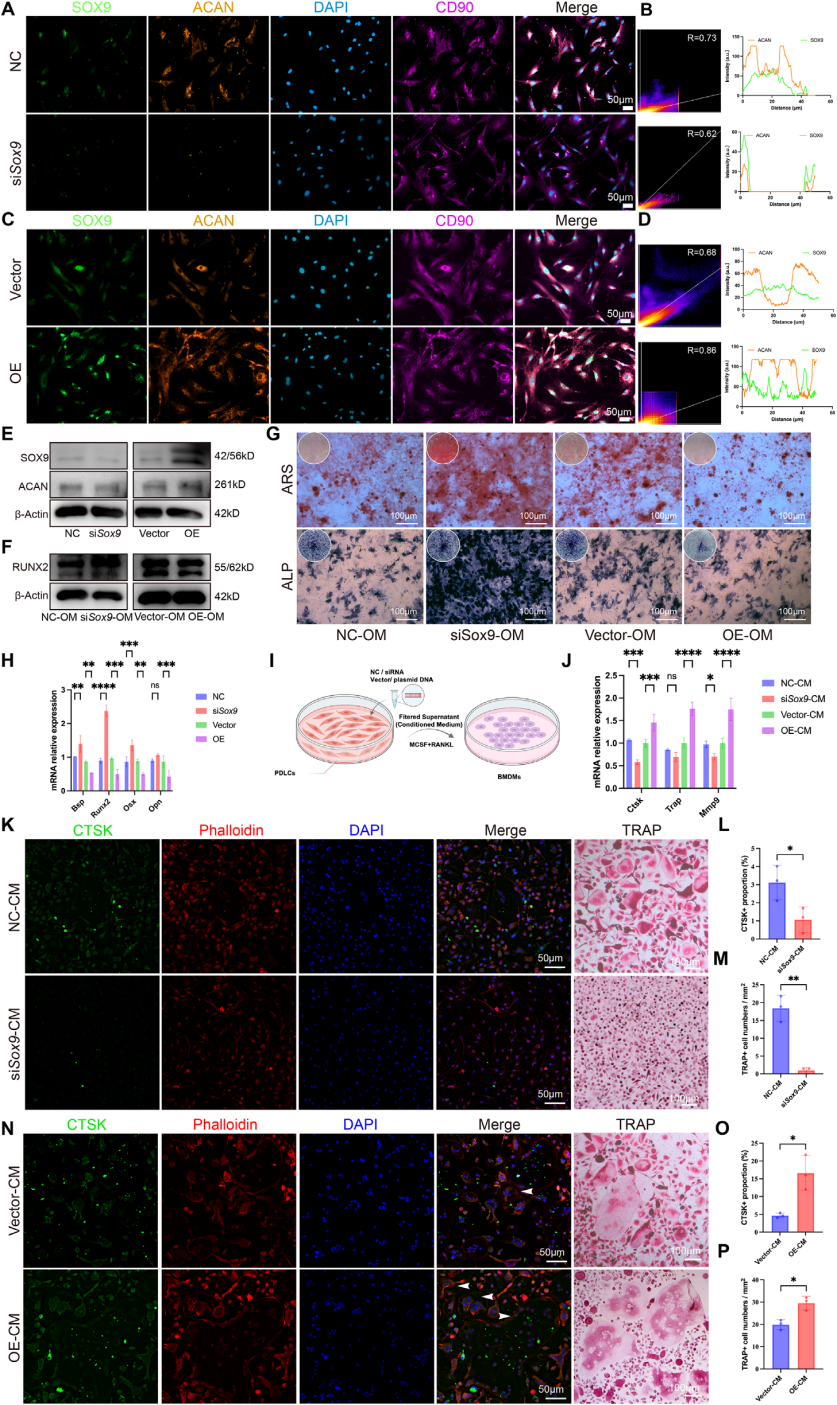
Sox9 modulates Sox9^+^Acan^+^ proportion within mouse PDLCs and osteoclastogenesis. (A–D) mIF showing SOX9, ACAN, and CD90 after Sox9 knockdown (si‐Sox9) or overexpression (OE), with colocalization quantification based on signal overlap. (E–F) Western blot validation of SOX9, ACAN, and RUNX2. (G) ALP and ARS staining under different Sox9 conditions, indicating altered osteogenic capacity. (H) Gene expression analysis of osteogenic markers. (I) Schematic of the conditioned‐medium culture. (J) RT‐qPCR analysis of osteoclast‐related genes. (K–P) IF of CTSK and TRAP staining showing osteoclast differentiation under NC‐CM, siSox9‐CM, Vector‐CM, and OE‐CM, with quantification of CTSK^+^ cells and TRAP^+^ osteoclasts. Scale bars are indicated. Data presentation and n: A–P, n = 3 independent biological experiments; values are shown as mean ± SD, and each dot represents one biological replicate. Statistics: Data are shown as mean ± SD. Statistical analysis was performed as described in the Methods section. Significance notation: ns, not significant (*P* ≥ 0.05); ^*^ *p* < 0.05; ^**^ *p* < 0.01; ^***^ *p* < 0.001; ^****^ *p* < 0.0001.

To investigate the functional role of *Sox9* and *Acan*, we performed knockdown (KD) and overexpression (OE) experiments in mouse PDLCs. Initial knockdown experiments showed that *Sox9* KD significantly reduced *Sox9* and *Acan* expression, while *Acan* KD yielded minimal effect on *Sox9* expression, consistent with *Sox9*'s role as an upstream regulator of *Acan* (Figure S5). Subsequent *Sox9* OE and *Sox9* KD efficiency was confirmed by Western blot (Figure 3E). Multiplex immunofluorescence (mIF) demonstrated that *Sox9* KD decreased, whereas *Sox9* OE increased the abundance of Sox9^+^Acan^+^ double‐positive cells, with high co‐localization confirmed by Pearson coefficients above 0.5 (Figure 3A–D). Functional assays revealed that *Sox9* KD enhanced osteogenic differentiation, as evidenced by increased mineralization in ALP and ARS staining (Figure 3G), elevated runt‐related transcription factor 2 (RUNX2) protein (Figure 3F), and upregulation of osteogenic genes, including *runt‐related transcription factor 2 (Runx2)*, *osterix (Osx)*, *bone sialoprotein (Bsp)*, and *osteopontin (Opn)* under excessive force (Figure 3H). Conversely, *Sox9* OE suppressed osteogenic differentiation. To assess paracrine effects on osteoclastogenesis, conditioned medium from manipulated mouse PDLCs was applied to bone marrow‐derived macrophages (Figure 3I). Conditioned medium from *Sox9* OE cells promoted osteoclast genes expression, including *Ctsk, acid phosphatase 5 (Trap)*, and *matrix metallopeptidase 9 (Mmp9)* (Figure 3J). It also increased osteoclast size, number, and multinucleation, as shown by CTSK/Phalloidin and TRAP staining (Figure 3N). These morphological changes corresponded with elevated CTSK^+^ and TRAP^+^ cell counts (Figure 3O,P). Conversely, medium from *Sox9* KD cells strongly inhibited osteoclast formation (Figure 3K–M).

### 2.4 FGF2–FGFR2 Signaling between Mesenchymal and Macrophage Populations under Mechanical Loading

**FIGURE 4:**
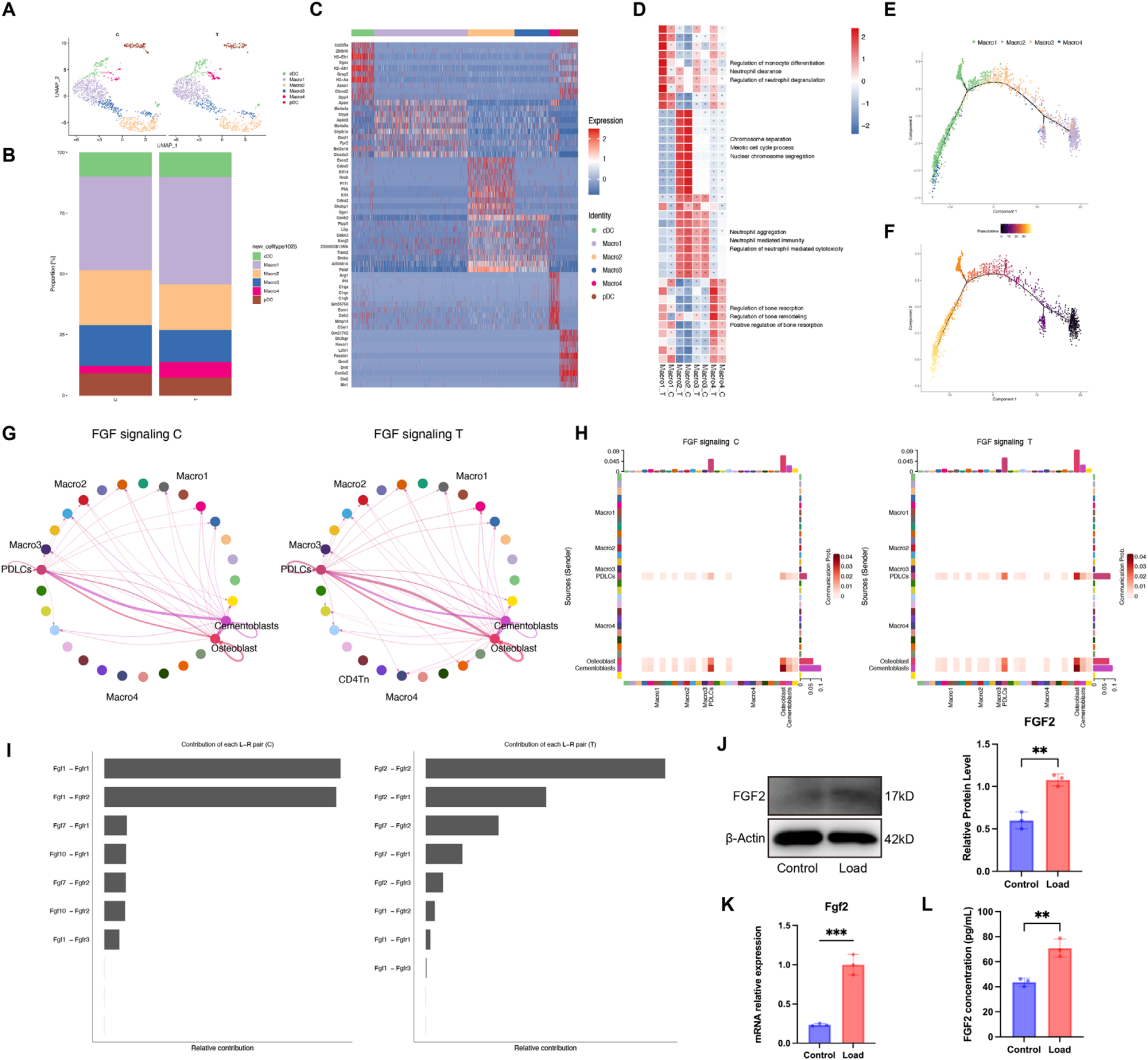
Single‐cell analysis reveals FGF–FGFR2 signaling axis. (A–F) Macrophage and dendritic cell analyses. (A) UMAP visualization of macrophage and dendritic cell populations. (B) Stacked bar plot showing the proportional composition of the four macrophage clusters. (C) Heatmap displaying canonical marker gene expression across macrophage clusters. (D) Barplot of the top 10 enriched functional pathways for each macrophage cluster. (E) Pseudotime trajectory of macrophage subpopulations inferred by monocle/trajectory analysis. (F) Pseudotime heatmap showing gene expression dynamics along the trajectory, with darker colors indicating early‐state expression and lighter colors representing late‐state expression. Macrophages and dendritic cells were analyzed together as they were co‐clustered within the myeloid lineage during initial classification. (G–I) CellChat analysis of the intercellular communication patterns mediated by the FGF signaling pathway in control (C) and loaded (T) groups. (G) Network graphs showing intercellular communication patterns mediated by FGF signaling. (H) Heatmaps depicting the interaction strength of FGF signaling between cell populations in the C and T groups. (I) Contribution plots showing the relative weights of individual ligand–receptor pairs within the FGF signaling pathway. (J–K) Western blot and RT‐qPCR validation showing increased FGF2 expression. (L) IF of FGF2 in PDL under force loading. ELISA quantification of FGF2 in cell‐culture supernatants collected from mouse PDLCs under loading conditions. Each biological replicate (n = 3) was measured in duplicate and averaged; data are shown as mean ± SD, with concentrations reported in pg/mL. Statistics: Data are shown as mean ± SD. Statistical analysis was performed as described in the Methods section. Significance notation: ns, not significant (*P* ≥ 0.05); ^*^ *p* < 0.05; ^**^ *p* < 0.01; ^***^ *p* < 0.001; ^****^ *p* < 0.0001.

To elucidate the mechanism underlying this pro‐osteoclastic effect, we re‐analyzed the macrophage population using dimensionality reduction and clustering with marker genes referenced in previous studies [28, 29]. Macrophages were divided into four subpopulations: Macro1 expressing *Apoe* and *Fpr2*, Macro2 enriched for *Kif4* and *Cdca2*, Macro3 marked by *Plpp5* and *Ccnb2*, and Macro4 distinguished by *Mmp14* and *C3ar1* (Figure 4A,C). The proportion of Macro4 increased from 3.01% in the control group to 6.37% in the treatment group (Figure 4B). Enrichment analysis indicated that Macro1 is associated with neutrophil degranulation, Macro2 with macrophage proliferation, Macro3 with neutrophil aggregation and immunity, and Macro4 with bone resorption and remodeling processes (Figure 4D). This subset was thus considered an osteoclast precursor macrophage population. Pseudotime analysis further confirmed that Macro4 represents a late‐stage differentiation state (Figure 4E,F). RNA velocity analysis of macrophage subsets further positioned Macro4 as the most terminally differentiated state (Figure S6).

CellChat analysis revealed a newly emergent fibroblast growth factor (FGF) signaling interaction between mouse PDLCs and Macro4 in the T group, as well as enhanced FGF communication between mouse PDLCs and osteoblasts (Figure 4G,H). Of the ligand–receptor pairs in the FGF pathway, FGF2–FGFR2 contributed most significantly (Figure 4I). An increase in receptor activator of nuclear factor‐κB ligand (RANKL) signaling between osteoblasts and Macro4 was also detected (Figure S6D). Although mouse PDLCs did not strongly interact with Macro4 via RANKL, potential crosstalk through non‐canonical pathways cannot be ruled out. Western blotting (WB) and reverse transcription quantitative PCR (RT‐ qPCR) assays validated increased FGF2 expression in mouse PDLCs under mechanical stress (Figure 4J,K). Additionally, mechanical loading resulted in increased FGF2 concentrations in the PDLC supernatant, as determined by ELISA (Figure 4L). Similarly, *Sox9* knockdown reduced FGF2 expression, whereas Sox9 overexpression increased it, as demonstrated by qRT‐PCR and ELISA (Figure S5I–L).

### 2.5 Experimental Validation of the FGF2–FGFR2 Signaling Axis

**FIGURE 5:**
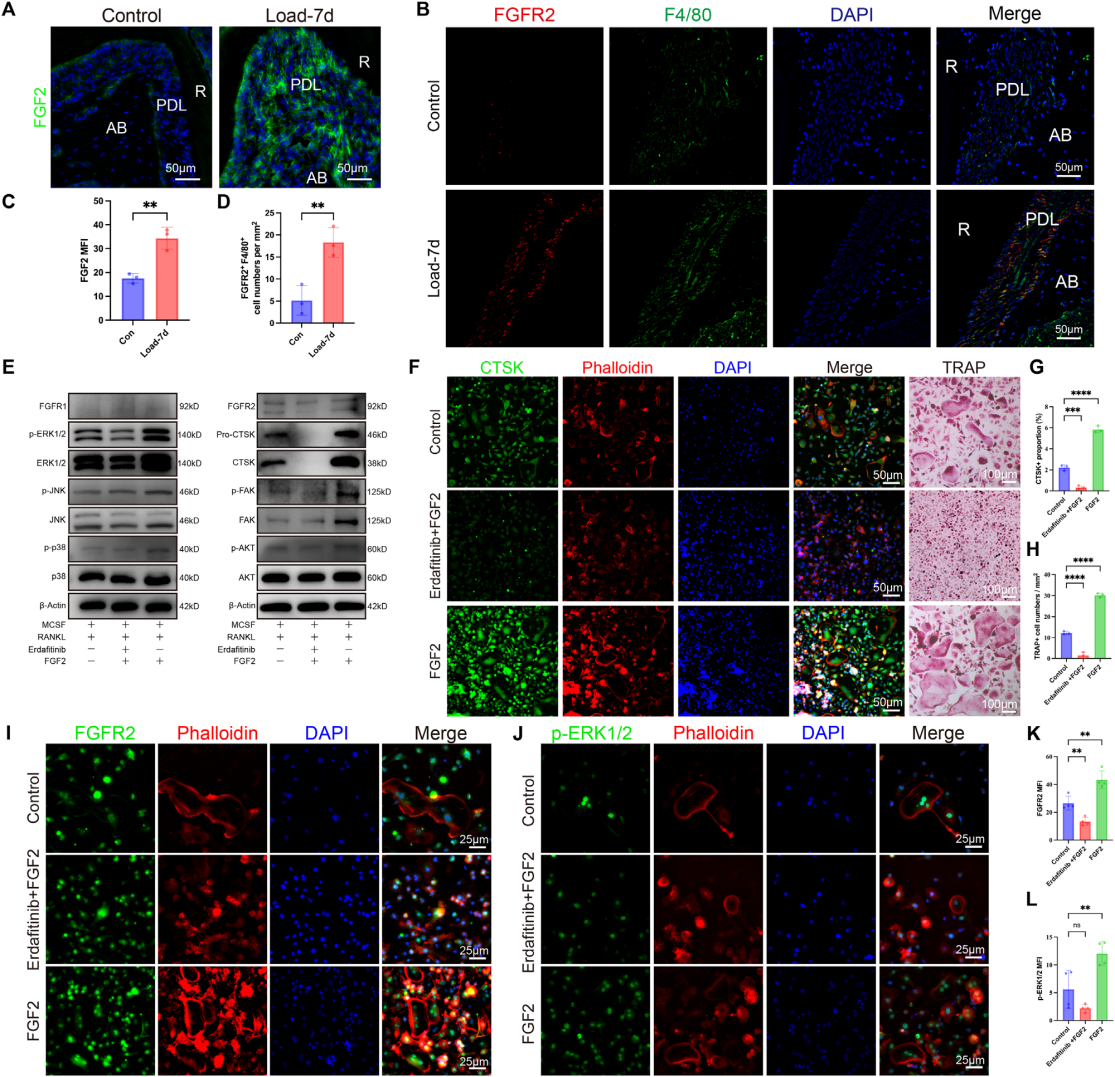
Functional Validation of the FGF2–FGFR2–ERK Axis. (A) Immunofluorescence staining of FGF2 in alveolar bone regions of mice with and without 7‐day orthodontic loading. (B) Immunofluorescence staining of FGFR2 and F4/80 under the same loading conditions. Anatomical labels: R, root; PDL, periodontal ligament; AB, alveolar bone. (C–D) Fluorescence quantification of FGF2. Double positive cell counts of FGFR2 and F4/80. (E) Western blot confirming FGF2–FGFR2–ERK signaling activation in vitro. (F–H) IF of CTSK/Phalloidin and TRAP staining showing osteoclast activity under FGF2 and inhibitor treatments, with quantification. (I–J) IF showing FGFR2 and p‐ERK1/2 expression under FGF2 stimulation and blockade. (K–L) Quantification of osteoclastogenesis. Scale bars are indicated. Statistics: Data are shown as mean ± SD. Statistical analysis was performed as described in the Methods section. Significance notation: ns, not significant (*P* ≥ 0.05); ^*^ *p* < 0.05; ^**^ *p* < 0.01; ^***^ *p* < 0.001; ^****^ *p* < 0.0001.

Our IF assay showed elevated FGF2 expression on the pressure side after 7 days of loading, accompanied by visibly increased colocalization of F4/80 and FGFR2 (Figure 5A,B). Quantitative analysis consistently confirmed higher FGF2 levels, along with a significant increase in F4/80^+^FGFR2^+^ double‐positive cells (Figure 5C,D). We then treated BMDMs with conditioned medium from mechanical force‐treated mouse PDLCs, with or without exogenous FGF2 and/or the FGFR inhibitor Erdafitinib. Public database screening suggested involvement of the MAPK and AKT serine/threonine kinase (AKT) pathways during osteoclast differentiation. Accordingly, key signaling molecules were probed, including extracellular signal‐regulated kinase (ERK), p38 mitogen‐activated protein kinase (p38), c‐Jun N‐terminal kinase (JNK), AKT, and focal adhesion kinase (FAK), as well as fibroblast growth factor receptor 1 (FGFR1) and FGFR2. WB confirmed corresponding changes in CTSK expression and strong activation of the ERK1/2 pathway, with minimal effect on other pathways (Figure 5E). Moreover, FGFR2 expression was moderately elevated by FGF2, while FGFR1 remained unchanged, aligning with CellChat predictions. IF and TRAP staining showed that FGF2 enhanced osteoclast differentiation, an effect blocked by Erdafitinib (Figure 5F–H). These results were further corroborated by IF staining, which showed increased FGFR2 and phospho‐ERK levels (Figure 5I–L). Thus, mechanical force induces a novel FGF2–FGFR2–ERK signaling axis between Sox9^+^Acan^+^ cells and Mmp14+ macrophages, specifically promoting osteoclastogenesis and alveolar bone remodeling.

### 2.6 Local Suppression of Sox9 Expression in Periodontal Mesenchymal Cells Mitigates Root Resorption

**FIGURE 6:**
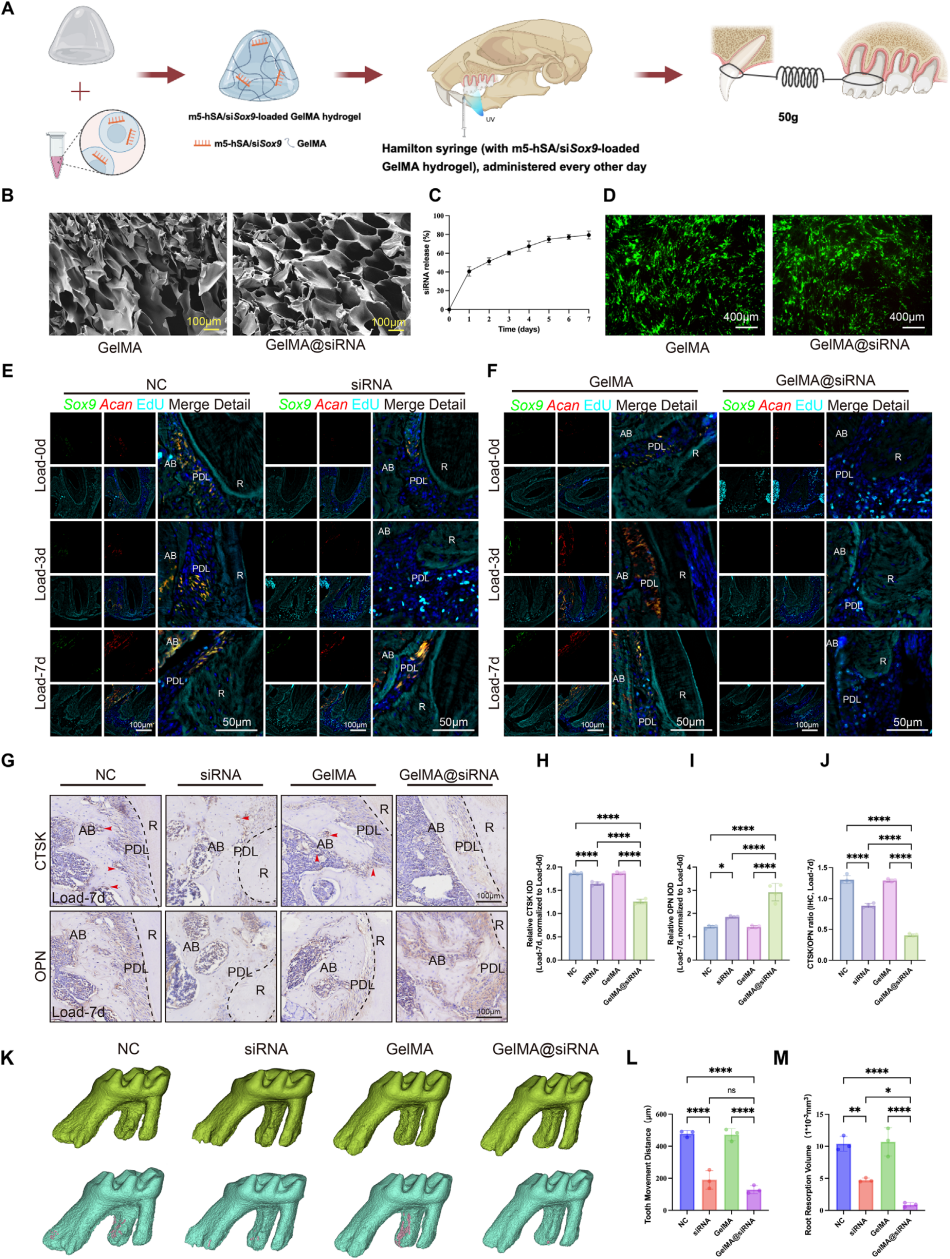
Local delivery of siRNA via GelMA hydrogel suppresses Sox9^+^Acan^+^ and attenuates root resorption. (A) Schematic illustration of the in vivo GelMA@siRNA delivery protocol, including periodontal injection, photocrosslinking based on established GelMA photopolymerization methods, alternate‐day dosing, and subsequent OTM force application. (B) SEM images showing the porous microstructure of GelMA@siRNA. (C) Cumulative release profiles of siRNA from GelMA over time (n = 7). (D) Live/dead staining showing good biocompatibility of GelMA@siRNA. (E–F) RNAscope of *Sox9*, *Acan*, and EdU after force loading, comparing NC, siRNA, GelMA, and GelMA@siRNA treatments. (G–J) IHC staining of CTSK and OPN in each group at load‐7d, with quantitative analysis normalized to the NC‐load‐0d baseline; red arrows indicate CTSK^+^ cells. Corresponding load‐0d IHC signals showed no significant differences among groups (Figure S8I–L). (K) MicroCT reconstruction showing root resorption under different treatments. (L–M) Quantification of OTM distance and root resorption area. Scale bars are indicated. Statistics: Data are shown as mean ± SD. Statistical analysis was performed as described in the Methods section. Significance notation: ns, not significant (*P* ≥ 0.05); ^*^ *p* < 0.05; ^**^ *p* < 0.01; ^***^ *p* < 0.001; ^****^ *p* < 0.0001.

Since direct isolation of this specific cell population was not feasible, we first attempted local injection of *Sox9*‐targeting siRNA administered every other day (Figure 6A). While this approach significantly reduced Sox9^+^Acan^+^ cell abundance at day 3, the effect was transient, with populations rebounding by day 7 (Figure 6E), indicating that bolus siRNA injection yielded an incomplete and unsustained knockdown. To overcome this limitation, we developed an injectable GelMA‐based hydrogel for sustained siRNA delivery (Figure 6B,C). The material demonstrated high biocompatibility in vitro (Figure 6D). We then compared four treatment groups: NC injection, siRNA, GelMA only, and GelMA@siRNA, all following the same injection schedule. Remarkably, only the GelMA@siRNA group achieved persistent suppression of Sox9^+^Acan^+^ cells throughout the 7‐day period without rebound (Figure 6F). This robust cellular knockdown translated into superior therapeutic efficacy. The GelMA@siRNA treatment markedly reduced CTSK and enhanced OPN expression at the compression side, indicating a shift toward lower resorptive activity and increased reparative responses (Figure 6G–J). Consistent directional changes were confirmed by dual‐marker mIF staining (Figure S8D–H). Micro‐CT analysis confirmed that GelMA@siRNA treatment markedly attenuated root resorption and delayed tooth movement, outperforming conventional siRNA injection (Figure 6K–M). Critically, the GelMA group showed no significant effect, confirming that the therapeutic benefits were attributable to sustained siRNA release rather than non‐specific effects of the hydrogel matrix. Taken together, these results demonstrate that direct targeting of the Sox9^+^Acan^+^ cell subpopulation via *Sox9* knockdown represents a viable and effective strategy for modulating orthodontic bone remodeling and mitigating root resorption.

## 3 Discussion

**FIGURE 7:**
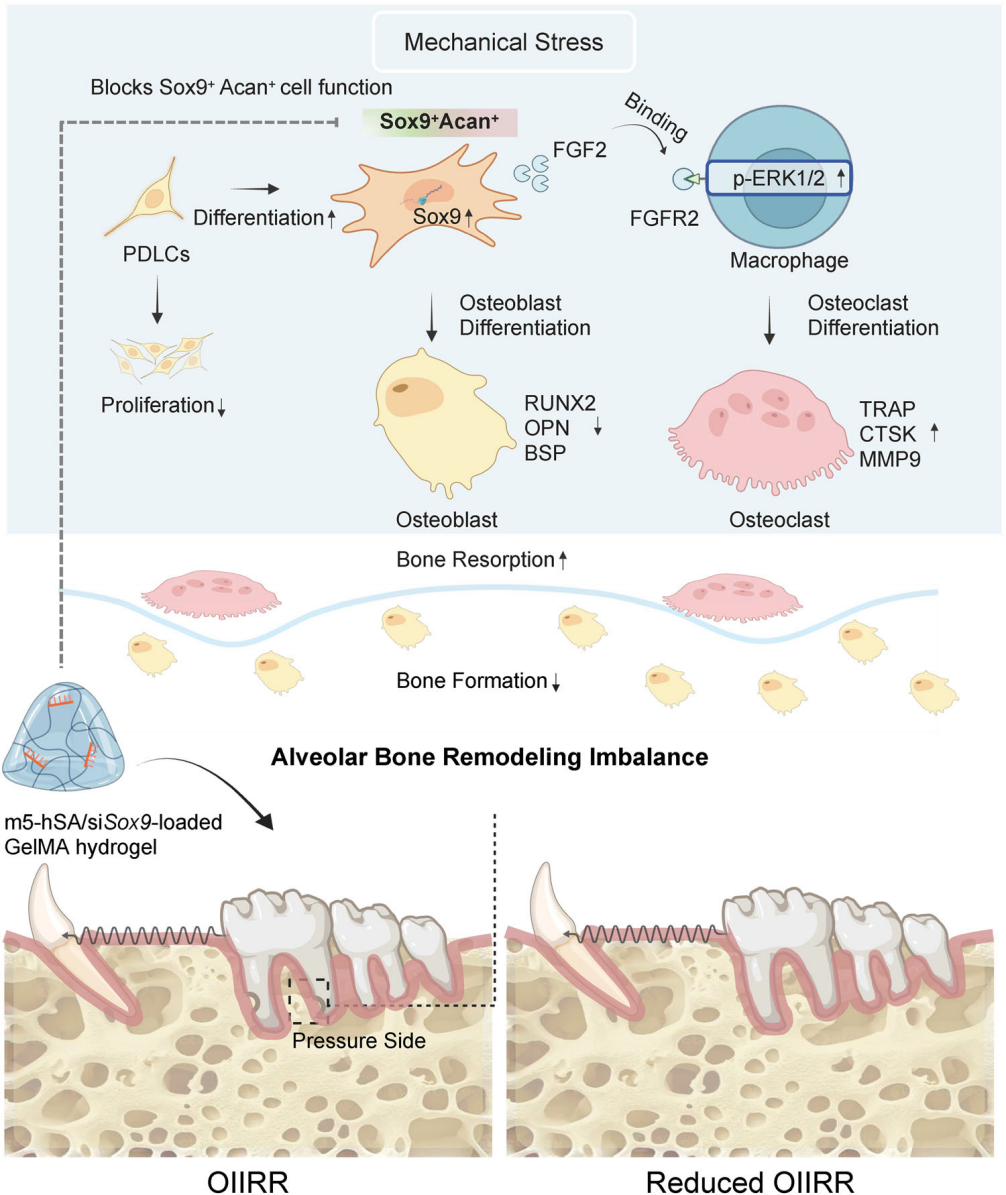
Schematic model illustrating the role of Sox9^+^Acan^+^ mesenchymal transitional cells in orthodontic tooth movement. Sox9^+^Acan^+^ mesenchymal transitional cells are activated by orthodontic force, respond via FGF–FGFR2–ERK signaling, and promote osteoclastogenesis through paracrine regulation. Local suppression of Sox9 by GelMA@siRNA effectively reduces osteoclast activity and alleviates root resorption, highlighting the translational potential of targeting mechanoresponsive stromal cell populations to achieve controlled orthodontic tooth movement.

Exploration of the early heterogeneity of periodontal mesenchymal lineage cells and their role in shaping subsequent immune responses during OTM addresses a critical gap in our understanding of OIIRR. The present study leverages an excessive‐force OTM model, a system that reliably generates pathological osteoclast overactivation and root resorption, to delineate the early mechanobiological events responsible for initiating OIIRR. Using single‐cell RNA sequencing, we identified a previously unrecognized force‐induced Sox9^+^Acan^+^ transitional mesenchymal subpopulation. This population plays a pivotal role in promoting osteoclastogenesis by activating the FGF2–FGFR2–ERK signaling axis. Building on these findings, we developed a GelMA@siRNA delivery system to locally suppress *Sox9* expression in the periodontal microenvironment. This intervention selectively attenuated the excessive osteoclast activation induced by mechanical loading while preserving the physiological bone remodeling required for effective tooth movement, resulting in reduced root resorption and improved periodontal protection (Figure 7). Collectively, these findings highlight the translational potential of precisely modulating mechanoresponsive stromal cell populations to achieve controlled tooth movement with minimal iatrogenic damage. Beyond orthodontics, this work illustrates how stromal intermediates integrate biomechanical cues with osteoimmune regulation, providing a conceptual link between local periodontal remodeling and broader principles of skeletal mechanobiology.

In this study, we identified a novel Sox9^+^Acan^+^ mesenchymal subpopulation within the periodontal ligament that emerges in response to mechanical loading and participates in bone remodeling. Although *Sox9* and *Acan* are widely recognized as canonical markers of chondrogenesis [30], extensive developmental studies have demonstrated that their expression is not restricted to cartilage. Sox9, in particular, plays broader roles in skeletogenesis by maintaining mesenchymal lineage stability and regulating multiple stages of chondrocyte differentiation [31]. Notably, Sox9 expression has also been observed in specific craniofacial and dental mesenchymal lineages, indicating a functional role that extends beyond cartilage. For example, Sox9^+^ progenitors have been identified not only in the hard palate but also in odontogenic regions [32]. Besides, Sox9^+^Msx1^+^ cells have been shown to function as key dental niche cells driving tooth formation [33]. In addition to the two loading‐associated scRNA‐seq datasets analyzed here, independent validation across multiple complementary datasets consistently revealed Sox9^+^Acan^+^ mesenchymal populations [34]. These datasets included human SHED cells and additional mouse periodontal ligament datasets, supporting the robustness of this transitional state across species and experimental contexts.

To further characterize this population, pseudotime analysis demonstrated that Sox9^+^Acan^+^ cells originate from mesenchymal stem cells and undergo lineage transitions in response to mechanical stimulation. These force‐induced trajectories reflect a broader biological principle whereby mechanical stress redirects mesenchymal stem cell fate decisions. Such plasticity is relevant not only to periodontal remodeling but also to fracture healing, skeletal development, and pathological bone loss. Previous studies have shown that lineage bias toward adipogenic rather than osteogenic commitment contributes to reduced bone mass and osteoporosis. In contrast, a Fat4^+^ population has been reported to initiate an alternative osteogenic pathway in alveolar bone [18]. Similarly, inflammatory conditions have been shown to drive CXCL12^+^ pericyte‐like mesenchymal cells into a pre‐osteoblastic state even after periodontal therapy [23]. Collectively, these findings indicate that Sox9^+^Acan^+^ cells constitute a mechanically induced, adaptive state of periodontal MSCs. This highlights a broader paradigm in which mechanoresponsive transitional states allow mesenchymal progenitors to fine‐tune tissue homeostasis across diverse skeletal contexts.

Crosstalk between mesenchymal lineage cells and immune cells is a central regulator of bone remodeling, mediated through both paracrine signaling and direct cell contact [35]. The FGF signaling axis is well recognized for its role in craniofacial and tooth development, in coupling between osteogenesis and osteoclastogenesis, and in maintaining periodontal tissue homeostasis [36]. FGF2, in particular, has been reported to exert dual roles: it promotes osteogenic differentiation through Wnt/β‐catenin pathways while also enhancing osteoclast activity by directly activating FGFR–MAPK (p42/44 ERK) signaling in mature osteoclasts, thereby contributing to the regulation of bone mass and osteoclastic gene expression [37]. Previous studies have implicated FGF2 in osteoclast formation [38, 39]. Immunohistochemical analyses have shown its upregulation on the pressure side in tooth movement models, supporting its functional relevance. Sox9^+^Acan^+^ cells drove osteoclastogenesis via FGF2 secretion, which activated the FGFR2–ERK pathway in Mmp14^+^ macrophages, thereby enhancing osteoclast formation. These findings reinforce a broader model in which mesenchymal cells act as mechanosensors that tune immune responses. Similar stromal–immune regulatory interactions have been described in bone marrow niches, joint microenvironments, and craniofacial sutures. For example, innate lymphoid cells interact with periodontal ligament cells during sterile inflammation in OTM [40], and PDL‐derived factors such as sclerostin regulate alveolar bone remodeling through osteocytic pathways [41]. Consistent with prior reports, CellChat analysis detected heightened RANKL–RANK signaling in the treatment group, confirming active osteoblast–macrophage crosstalk [42, 43]. Collectively, the above findings identify a paracrine mechanism in which force‐induced mesenchymal cells promote osteoclastogenesis through macrophage ERK activation. This provides a case study of how mechanical stress orchestrates stromal–immune partnerships in skeletal biology.

Cellular and molecular events at early stages critically shape subsequent outcomes [44]. A 1‐day post‐loading time point was selected, following strategies used in other bone‐related conditions. Research across skeletal pathologies reveals the importance of early‐phase mechanisms: the inflammatory trigger in fracture healing [45, 46], the subchondral changes driving osteoarthritis [47], and the rapid osteoclast activation in osteoporosis [48, 49]. Likewise, focusing on this early window enables dissection of core mechanisms underlying OIIRR initiation.

Achieving controlled tooth movement while minimizing adverse effects remains a significant challenge in orthodontic practice [6]. Current strategies for modulating tooth movement and root resorption each have important limitations. Systemic drug administration lacks specificity and causes off‐target effects [50]. Local injections provide poor retention and require repeated trauma [51]. Physical approaches result in only mild and non‐specific outcomes [52]. To address these issues, we developed a GelMA@siRNA sustained‐release system that offers a targeted and mechanistically informative approach. Inspired by previous studies showing that GelMA hydrogels can be applied for localized and sustained siRNA delivery, we adopted a similar strategy by loading chemically modified m5‐hSA/si*Sox9* into GelMA to achieve controlled release in the periodontal microenvironment [53, 54]. This system leverages siRNA‐mediated silencing to achieve high specificity, selectively modulating *Sox9* expression or Sox9^+^Acan^+^ cell populations. The hydrogel matrix ensures safety and localization by confining the therapeutic agent, thereby minimizing systemic exposure. Following in situ photopolymerization, the GelMA network provides sustained release over several days, offering a stable and effective local delivery window [55, 56, 57]. Besides, from a therapeutic perspective, root resorption occurs primarily during active mechanical loading and diminishes once periodontal tissues recover. This indicates that prolonged gene suppression is not required for therapeutic benefit. Taken together, these findings demonstrate how engineered biomaterials can be leveraged to modulate force‐responsive mesenchymal populations in vivo, with potential implications extending beyond orthodontics to regenerative strategies in orthopedics and craniofacial medicine.

Nevertheless, several limitations should be noted. Direct isolation of Sox9^+^Acan^+^ cells and lineage‐resolved functional assays are essential to define their specific roles. The regulatory relationship between Sox9 and FGF2 requires further clarification, particularly whether Sox9 exerts direct transcriptional control, which could be assessed through ChIP or reporter assays. The role of FGFR2 in macrophage‐mediated osteoclastogenesis requires further investigation, and macrophage‐specific loss‐of‐function models will clarify the FGF2–FGFR2–ERK axis specificity. Although GelMA@siRNA enables spatially restricted delivery, its lack of cell‐type specificity underscores the need for more refined targeting systems. Future strategies should focus on developing cell‐type‐specific delivery platforms, such as GelMA‐based systems, to enhance therapeutic precision. The modest reduction in tooth displacement reflects the clinical challenge of suppressing pathological remodeling while preserving physiological function. Further studies are needed to consolidate these mechanistic insights and support safe, clinically translatable applications of this approach.

## 4 Conclusion

In summary, we delineate a force‐induced Sox9^+^Acan^+^ transitional mesenchymal cell population that promotes osteoclastogenesis through activation of the FGF2–FGFR2–ERK signaling pathway during orthodontic tooth movement. Conceptually, this work identifies a mechanoresponsive stromal intermediate that integrates mechanical cues with osteoimmune regulation, extending current paradigms of periodontal and skeletal mechanobiology. Clinically, the GelMA@siRNA platform enables localized, transient Sox9 suppression. This provides a practical strategy to inhibit pathological osteoclast overactivation and mitigate root resorption without compromising tooth movement.

## 5 Experimental Section

### 5.1 Animals and Ethical Approval

One hundred male C57BL/6J mice aged 6–8 weeks were obtained from GemPharmatech (Nanjing, China). They were maintained in a specific pathogen‐free (SPF) facility under a 12‐h light/dark cycle with unrestricted access to chow and water. After a one‐week acclimatization period, 90 mice were randomly divided into 15 groups (n = 6 per group) based on two variables, namely the duration of force application (1, 3, or 7 days) and the type of local intervention. The intervention groups included force‐only (OTM), NC treatment, siRNA treatment, GelMA hydrogel implantation, and GelMA@siRNA delivery. In all animals, the orthodontic appliance was unilaterally placed on the maxillary right first molar to establish the OTM model, while the contralateral left side served as the internal control. No separate control animals were established. Moreover, another 10 mice were used for single‐cell RNA sequencing (scRNA‐seq). In these animals, the maxillary right first molar was subjected to orthodontic force for 24 h, while the left side remained untreated as the control. Periodontal tissues from both sides were harvested for scRNA‐seq analysis. All experimental procedures were reviewed and approved by the Ethics Committee of the College of Stomatology, Chongqing Medical University (CQHS‐REC‐2024(LSNo. 162)) and conducted in accordance with the Animal Research: Reporting of *In Viv* *o* Experiments (ARRIVE) guidelines.

### 5.2 Establishment of the OTM Model

OTM was induced by placing a nickel–titanium closed‐coil spring (50 g, TOMY Sentalloy, TOMY Inc., Japan) between the maxillary right first molar and the ipsilateral incisor. This high‐force magnitude is widely recognized to induce excessive mechanical stress and reproducibly trigger OIIRR. The spring was fixed with a 0.1 mm stainless‐steel ligature wire and light‐cured composite resin, and the applied force was verified with a tension gauge. Appliance integrity and mouse welfare were monitored daily. After the designated force application periods, euthanasia was performed using CO_2_ exposure, immediately followed by cervical dislocation.

### 5.3 Tissue Collection and Single Cell Suspension

After euthanasia, periodontal tissues surrounding the maxillary right first molar and the adjacent alveolar bone within 1 mm on the buccal and palatal sides were carefully dissected, with the gingiva removed. For scRNA‐seq, tissues from 10 mice per condition (control and 1‐day force) were pooled. Samples were minced and digested in HBSS (Gibco; Thermo Fisher Scientific, Cat. No. 14025092, USA) containing Collagenase P (Sigma–Aldrich, Cat. No. 11213857001, USA) and Dispase II (Sigma–Aldrich, Cat. No. D4693‐1G, USA) at 37°C for 30 min with gentle agitation. The reaction was terminated with 10% FBS (ExCell Bio, Cat. No. FSP500, Shanghai, China), and the suspension was filtered sequentially through 70 and 40 µm strainers. Cells were pelleted at 300 g for 5 min, resuspended in phosphate buffer saline (PBS; Solarbio, P1003‐2 L, Beijing, China) containing 0.04% BSA (Sigma–Aldrich, Cat. No. A9418, USA), and cell viability was assessed by Trypan Blue exclusion (Thermo Fisher Scientific, Cat. 15250061, USA). All suspensions showed >95% cell viability.

### 5.4 Single Cell Library Preparation and Sequencing

Single‐cell suspensions (700–1200 viable cells/µL) were loaded onto the 10x Genomics Chromium Controller. Libraries were constructed using the Chromium Single Cell 3′ Reagent Kit v3.1 and Chromium Single Cell G Chip Kit (10x Genomics), following the manufacturer's protocol. Reverse transcription and barcoding were performed in GEMs, followed by cDNA amplification and library construction. Libraries were sequenced on an Illumina NovaSeq 6000 (Illumina, USA) using 28‐bp Read1, 8‐bp i7 index, and 91‐bp Read2, yielding a median of ∼30 000 reads per cell after filtering.

### 5.5 Single Cell Bioinformatic Analysis

Raw sequencing data were processed with Cell Ranger (v8.0.1, 10x Genomics) using the mouse reference genome (GRCm39‐2024‐A) to generate gene‐barcode matrices. Cells with low gene counts, high mitochondrial transcript percentage, or extreme Unique Molecular Identifier (UMI) counts were excluded. Downstream analyses were performed in Seurat (normalization, scaling, PCA, and clustering with t‐SNE/UMAP). Cluster annotation was based on canonical marker genes. Macrophages and dendritic cells were jointly included in the secondary clustering because they emerged from the same myeloid lineage cluster during the initial analysis and displayed overlapping transcriptional profiles. Pseudotime trajectories were reconstructed with Monocle 3, and differentially expressed genes were subjected to functional enrichment and pathway analyses.

For scRNA‐seq–based analyses, including UMAP visualization, pseudotime trajectory, RNA velocity, and gene set enrichment analysis (GSEA), data are presented descriptively, and no formal statistical testing was applied unless otherwise stated.

### 5.6 Public Dataset Validation

In addition to our own dataset, we incorporated GSE287729 (released on GEO in February 2025), which examined OTM in C57BL/6J mice. For this model, a nickel–titanium coil spring generating 25 g of force was fixed between the upper right first molar and the incisors, and tissues were harvested at day 7. To enrich the affected microenvironment, alveolar bone adjacent to the extraction site (≈ 1 mm margin) was collected from six animals per group and pooled. Samples were enzymatically digested with a commercial dissociation system optimized for bone, followed by removal of red blood cells under controlled temperature conditions. The resulting single‐cell suspensions were subjected to high‐throughput scRNA‐seq on an Illumina HiSeq X Five platform. After standard quality filtering and normalization, we applied the same marker‐guided cell type annotation used for our in‐house dataset to evaluate the representation of the Sox9^+^Acan^+^ subpopulation. Two previously published periodontal scRNA‐seq datasets (GSE160358 and GSE168450) were also processed under this unified pipeline for comparative validation. To extend validation across species, we further incorporated the human SHED scRNA‐seq dataset GSE221216. This dataset contains single‐cell transcriptomes from stem cells of human exfoliated deciduous teeth and provides an independent human‐derived reference.

### 5.7 Micro‐CT and Histology

Maxillae (n = 90) were dissected and fixed in 4% paraformaldehyde (PFA; Servicebio, G1101‐500ML, China) at 4°C for 24 h. Micro‐CT imaging was performed with a Scanco µCT 50 system (Scanco Medical, Switzerland) under the following parameters: 10 µm voxel size, 55 kVp, 145 µA, and 300 ms integration time. 3D reconstructions were then analyzed in Mimics 21.0 to measure mesial displacement of the first molar as well as root resorption volume. After scanning, specimens were decalcified in 10% ethylenediaminetetraacetic acid (EDTA; Elabscience, Cat. No. E‐IR‐R112, China), pH 7.4, at 4°C with gentle agitation, dehydrated through graded ethanol, cleared in xylene, and embedded in paraffin (FFPE). Coronal sections (5 µm) were stained with hematoxylin and eosin (H&E) for general morphology or with tartrate‐resistant acid phosphatase (TRAP; Servicebio, Cat. No. G1050, China) for osteoclast detection. The phase‐contrast inverted microscope (Olympus, Japan) was used to conduct observations and obtain photographs.

### 5.8 RNAscope In Situ Hybridization

Tissues were fixed in 10% neutral‐buffered formalin at room temperature for 16–24 h. After fixation, the tissues were dehydrated through a series of graded ethanol solutions, cleared in xylene, and then embedded in paraffin. The tissues were sectioned into 5 µm slices using a microtome (Leica Biosystems, Germany), transferred to microscope slides, and dried at 60°C for 30 min on a slide warmer. Dewaxing was followed by a 10‐min incubation in pretreatment solution A at room temperature to remove endogenous enzymes, followed by boiling in citrate‐containing pre‐reaction solution B for 15 min. To expose mRNA, sections were digested with protease at 40°C for 15 min, then incubated with a gene‐specific probe in hybridization solution at 40°C for 2 h. After hybridization, unhybridized probes were washed off, and signal amplification was performed using a cascade signal amplification technique based on nucleic acid‐protein hybridization. A fast red substrate was added to an alkaline phosphatase‐based color reaction, revealing the target RNA as red or green dots or patches. The mRNA in situ detection for each gene was performed manually using the PinpoRNA RNA in situ hybridization detection kit (GD Pinpoease, Cat. No. PIF2000, China). The probes targeted the 1662‐3760 base region of *Sox9* (GD Pinpoease, Cat. No. 206821‐B2, China) and the 1705‐2820 base region of *Acan* (GD Pinpoease, Cat. No. 115951‐B1, China). After completion of all probe labeling, EdU staining was subsequently performed using the Cell‐Light EdU Apollo647 In Vitro Imaging Kit (Beyotime, Cat. No. C0071S, China) according to the manufacturer's instructions to detect proliferating cells. Finally, nuclei were counterstained with Hoechst 3342 (Beyotime, Cat. No. C1028, China). Hoechst was preferred to DAPI as a nuclear counterstain due to its reduced non‐specific binding to RNA, which optimizes the signal‐to‐noise ratio for the precise visualization and quantification of RNA puncta. Images were captured as described in the Microscopy section and quantified as described in the Image analysis section.

### 5.9 Multiplex Immunohistochemistry (mIHC)

mIHC was performed on formalin‐fixed, paraffin‐embedded (FFPE) sections of mouse maxillae using the Panovue mIHC Kit (Panovue, Cat. No. 10079100020, China), following the manufacturer's protocol. Briefly, after deparaffinization and antigen retrieval, endogenous peroxidase activity was quenched, and sections were blocked with commercial blocking buffer. Primary antibodies were applied sequentially according to a pre‐optimized staining order (Table S2). HRP‐conjugated secondary antibodies and tyramide signal amplification were used to develop the signal. Between staining cycles, bound antibodies were stripped using microwave treatment to avoid cross‐reactivity. Finally, nuclei were counterstained with DAPI (Beyotime, Cat. No. C1028, China), and slides were mounted in antifade medium. Images were captured as described in the Microscopy section and quantified as described in the Image analysis section.

### 5.10 Immunofluorescence (IF)

IF and multiplex immunofluorescence (mIF) staining were performed on paraffin‐embedded p tissue sections following standard protocols. Briefly, sections were deparaffinized, rehydrated, and subjected to antigen retrieval, then blocked and incubated with primary and fluorophore‐conjugated secondary antibodies. For mIF, sequential rounds of staining and signal development were conducted to visualize multiple markers within the same section. Images were captured as described in the Microscopy section and quantified as described in the Image analysis section.

### 5.11 Microscopy

All fluorescence images were acquired using a Leica DM6 B upright fluorescence microscope (Leica Microsystems, Germany). Acquisition parameters, including objective magnification, exposure time, and filter settings, were kept constant within each experiment. Confocal images for RNAscope were acquired using a confocal microscope (Leica TCS SP8, Germany), using identical laser and detector settings across samples. Representative images were processed uniformly in FIJI/ImageJ v1.53t (Fiji Is Just ImageJ; National Institutes of Health, Bethesda, USA) without altering the original signal intensity.

### 5.12 Image Analysis

Image quantification was performed in QuPath (version 0.4.3, University of Edinburgh, UK) and FIJI. Positive cell detection, ROI annotation, and intensity measurements followed pre‐defined thresholds and batch processing settings that were kept constant across groups. For spatial metrics, custom Python scripts were used to compute relative distances and the average number of positive cells per ROI.

### 5.13 Isolation and Culture of Mouse PDLCs and BMDMs

After euthanasia, mouse heads were surface‐sterilized with 75% ethanol, and maxillae and mandibles were dissected. First molars were extracted, minced into 1–2 mm^3^ fragments, and digested with Collagenase P and Dispase II at 37°C (800 rpm, 15 min). Digestion was neutralized with FBS, and cells were pelleted, washed, and plated, with flasks inverted and subsequently placed upright after 8 h. Colony‐forming unit fibroblast (CFU‐F) assay and flow cytometry (CD90^+^, CD73^+^, CD105^+^, CD45^−^, CD34^−^, CD14^−^) confirmed the identity of mouse PDLCs.

For BMDMs, bone marrow cells were isolated from femurs and tibias of wild‐type C57BL/6J mice by flushing with cold RPMI‐1640 medium (Gibco; Thermo Fisher Scientific, Cat. No. 11875093, USA), followed by filtration and centrifugation. PDLCs were cultured in MesenCult Expansion Kit (Mouse) (STEMCELL Technologies, Cat. No. 05513, Canada). BMDMs were cultured in complete RPMI‐1640 supplemented with 10% FBS, 1% penicillin‐streptomycin (PS; HyClone, SV30010, USA), and 70 ng/mL recombinant M‐CSF (PeproTech, Cat. No. 315‐02, USA) at 37°C with 5% CO_2_. The medium was refreshed every 3 days, and adherent macrophages were harvested after 7 days of differentiation for downstream experiments.

### 5.14 In Vitro Mechanical Loading and Osteogenic Differentiation

Cells were subjected to 2 g/cm^2^ static compression for 12 h to promote osteogenic differentiation. This loading regimen was delivered via a sterile glass weight placed on a sterile coverslip, and control groups remained unloaded. Flow cytometric analysis via Annexin V/PI (Annexin V‐FITC/PI Apoptosis Detection Kit; Beyotime, Cat. No. C1062M, China) was performed to confirm that no significant apoptosis was induced, ensuring that subsequent differentiation effects were not attributable to cellular stress or death. Following loading, cells were cultured in osteogenic induction medium consisting of α‐MEM (Gibco; Thermo Fisher Scientific, Cat. No. 12571‐048, USA) supplemented with 50 µg/mL ascorbic acid (MCE, Cat. No. HY‐B0166, China), 10 mm β‐glycerophosphate (MCE, Cat. No. HY‐126304, China), and 100 nm dexamethasone (MCE, Cat. No. HY‐14648, China). Differentiation was evaluated by ALP staining on day 7 using a BCIP/NBT Alkaline Phosphatase Color Development Kit (Beyotime, Cat. No. C3206, China) and by ARS staining on day 21 (Alizarin Red S Staining Kit; Cyagen, Cat. No. RASMX‐90021, China).

### 5.15 Western Blotting (WB) and Reverse Transcription Quantitative PCR (RT‐qPCR)

Total protein was extracted by the RIPA lysis buffer (Beyotime, P0013B/100 mL, China), and the BCA protein assay kit (Beyotime, Cat. No. P0012, China) was utilized to ascertain the concentration of the protein extracted. Proteins were separated by sodium dodecyl sulfate–polyacrylamide gel electrophoresis (SDS–PAGE) using SurePAGE precast polyacrylamide gels (Yeasen Biotechnology, Cat. No. 20310ES10, China) and subsequently transferred onto a polyvinylidene fluoride (PVDF) membrane (Immobilon‐P, 0.2 µm pore size; Merck Millipore, Cat. No. IPVH00005, Germany). Target proteins were detected using specific primary antibodies (Table S2). RNA was extracted and reverse transcribed into cDNA. RT‐qPCR was conducted with SYBR Premix (QIAGEN, Cat. No. 1129280, Germany). β‐actin was used for normalization, and relative transcript levels were calculated by the 2^−^ΔΔCt method. Primer sequences are provided in Table S3.

### 5.16 Gene Transfection and Knockdown

*Sox9* expression was silenced with siRNA (Lipofectamine 3000; Thermo Fisher Scientific, Cat. No. L3000015, USA) or overexpressed using a *Sox9* plasmid; controls received scrambled siRNA or empty vector (Tables S4 and S6). Knockdown or overexpression efficiency was verified by qRT‐PCR and WB.

### 5.17 Preparation of Conditioned Medium (CM)

CM was collected from transfected or mechanically loaded mouse PDLCs, centrifuged to remove debris, and filtered through a 0.22‐µm membrane. The resulting CM was stored at 4°C for short‐term use or aliquoted at –80°C for subsequent experiments.

### 5.18 Induction of Osteoclastogenesis in BMDMs

BMDMs from wild‐type mice were pre‐cultured with M‐CSF (70 ng/mL) for 3 days and subsequently induced with M‐CSF and RANKL (PeproTech, 100 ng/mL) in the presence of 50% PDLC‐derived CM for 5–7 days. Additional groups were treated with recombinant FGF2 (75 ng/mL), the FGFR inhibitor Erdafitinib (1 nm), or both (Figure S7C,D). Osteoclast differentiation was assessed by TRAP staining, CTSK immunofluorescence, and F‐actin ring formation visualized with phalloidin. For quantitative analyses, osteoclasts were defined as multinucleated (≥ 3 nuclei) TRAP‐positive cells.

### 5.19 Material Characterization

0.25% (w/v) LAP and gelatin methacryloyl (GelMA; Yongqinquan Intelligent Equipment Co., Ltd., EFL‐GM‐60, China) were mixed for 30 min at 70°C until completely dissolved to obtain a 20% (w/v) GelMA‐hybrid hydrogel. GelMA@siRNA hydrogels were assessed for release behavior, cytocompatibility, and morphology. For the release assay, constructs were incubated in RNase‐free PBS (pH 7.4) at 37°C in the dark, and 1 mL of medium was sampled at set intervals with equal replenishment. FITC fluorescence was detected in black 96‐well plates (Corning 3603, clear bottom, TC‐treated; Corning, Cat. No. 3603, USA) using a plate reader (BioTek Synergy H1, USA; Ex/Em 485/528 nm), and concentrations were determined from a standard curve (0–1000 nm, R^2^ > 0.99). Results were adjusted for sampling and expressed as cumulative release relative to the initial siRNA load. Biocompatibility was examined by culturing periodontal ligament stem cells on hydrogels for 48 h, followed by calcein‐AM/propidium iodide staining and imaging with a Leica DM6 B upright fluorescence microscope. For field‐emission scanning electron microscope (SEM) analysis, hydrogels were fixed in 2.5% glutaraldehyde, dehydrated in graded ethanol, sputter‐coated with gold (Quorum Q150R ES, Quorum Technologies, UK), and visualized using a Hitachi SU‐8010 field‐emission SEM (Hitachi High‐Technologies, Japan) at 5 kV. Rheological performance (Anton Paar, Austria) was characterized using a rotational rheometer (Figure S7A,B).

### 5.20 In Vivo EdU and siRNA Hydrogel Delivery

To evaluate the spatial distribution and proliferation of Sox9^+^Acan^+^ cells in vivo, 5ethynyl2′‑deoxyuridine (EdU; Invitrogen Click‑iT EdU, Thermo Fisher Scientific) was injected at a dose of 5 mg/kg via intraperitoneal injection on Day 0, prior to the initiation of orthodontic tooth movement (OTM). EdU is a thymidine analog that incorporates into newly synthesized DNA during S‐phase and can be detected using click chemistry methods. EdU injections were repeated every other day throughout the experiment to assess cell proliferation in the periodontal ligament.

Prior to all procedures, the animals were anesthetized using isoflurane (2% for induction, 1.5% for maintenance) in oxygen. Following EdU injection, 5 µL of GelMA hydrogel mixed with m5‐hSA/siSox9 to form GelMA@siRNA (or GelMA alone as control) was injected into the periodontal ligament around the maxillary first molar using a Hamilton syringe (Hamilton Company, USA). The hydrogel was then subjected to photocrosslinking with 365–405 nm light for 30 s, according to established GelMA photopolymerization protocols [53, 54, 55]. OTM was initiated immediately after the crosslinking process. The treatment was administered before the establishment of OTM models to achieve early and sustained intervention, with injections repeated every other day to maintain local siRNA availability.

### 5.21 Graphical Illustration

All schematic illustrations in this study were drawn using BioRender.

### 5.22 Statistical Analysis

Data were presented as mean ± standard deviation. For two‐group comparisons, a Student's *t*‐test was used. For multiple group comparisons, a one‐way or two‐way analysis of variance (ANOVA) with Tukey post hoc tests was performed using GraphPad Prism 10.0 (USA). Significance notation: ns, not significant (*P* ≥ 0.05); ^*^ *p* < 0.05; ^**^ *p* < 0.01; ^***^ *p* < 0.001; ^****^ *p* < 0.0001.

## Author Contributions

Miao Tan conceived and designed parts of the study together with Jie Li and Lan Huang, performed the majority of experiments, conducted data analysis, and drafted the manuscript. Minyu He, Mingrui Zong, Qiya Tang, Yinan Liu, Jiaju Deng, Shun Huang, and Xiaoxiao Lei assisted with experiments and data acquisition. Jie Li and Lan Huang supervised the project and revised the manuscript, and Lan Huang provided the majority of funding support. All authors contributed to manuscript editing and approved the final version.

## Conflicts of Interest

The authors declare no competing interests.

## Supporting information




**Supporting File**: advs73951‐sup‐0001‐SuppMat.pdf.

## Data Availability

The scRNA‐seq data generated in this study have been deposited in the NCBI Gene Expression Omnibus (GEO) under accession number GSE309282. The data are currently under controlled access for peer review and will be made publicly available upon publication.
