# Supplementary material for: Identification of a Force‐Induced Sox9+Acan+ Transitional Subpopulation Linked to FGF2–FGFR2–ERK Signaling in Orthodontic Bone Remodeling
Source: Adv Sci (Weinh). 2026 Jan 22;13(18):e19330. doi: 10.1002/advs.202519330 (PMC13042456; doi:10.1002/advs.202519330)
Supplement: Supplementary file 1 — Supporting File: advs73951‐sup‐0001‐SuppMat.pdf. [file ADVS-13-e19330-s001.pdf]

Identification of a Force-Induced Sox9$^+$Acan$^+$ Transitional Subpopulation Linked to FGF2–FGFR2–ERK Signaling in Orthodontic Bone Remodeling

Miao Tan[1,2,3,4], Minyu He[1,2,3,4], Mingrui Zong[1,2,3,4], Qiya Tang[1,2,3,4], Yinan Liu[1,2,3,4], Jiaju Deng[1,2,3,4], Shun Huang[1,2,3,4], Xiaoxiao Lei[1,2,3,4], Jie Li[1,2,3,4,], Lan Huang[1,2,3,4,]**

[1]College of Stomatology, Chongqing Medical University, Chongqing, 401147, PR China

[2]Chongqing Key Laboratory of Oral Diseases, Chongqing Medical University, Chongqing, 401147, PR China

[3]Chongqing Municipal Key Laboratory of Oral Biomedical Engineering of Higher Education, Chongqing Medical University, Chongqing, 401147, PR China

[4]Chongqing Municipal Health Commission Key Laboratory of Oral Biomedical Engineering, Chongqing Medical University, Chongqing, 400017, PR China

E-mail: jieli@hospital.cqmu.edu.cn; lanhuang@hospital.cqmu.edu.cn

Supplementary Figures

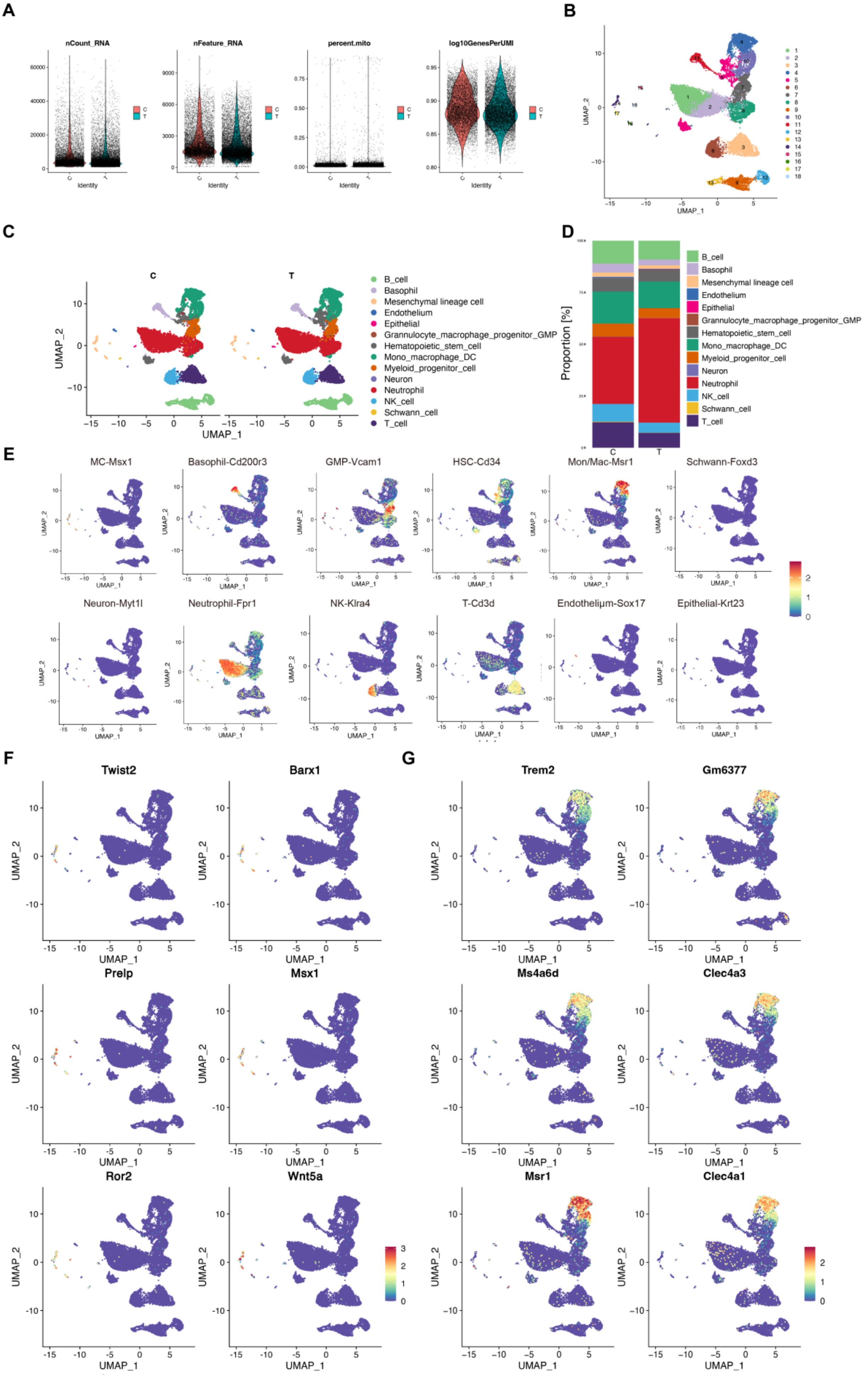
A
nCount_RNA
nFeature_RNA
percent.mito
log10GenesPerUMI
Identity
B
UMAP_1
UMAP_2
C
T
B_cell
Basophil
Mesenchymal lineage cell
Endothelium
Epithelial
Granulocyte_macrophage_progenitor_GMP
Hematopoietic_stem_cell
Mono_macrophage_DC
Myeloid_progenitor_cell
Neuron
Neutrophil
NK_cell
Schwann_cell
T_cell
D
Proportion [%]
E
MC-Msx1
Basophil-Cd200r3
GMP-Vcam1
HSC-Cd34
Mon/Mac-Msr1
Schwann-Foxd3
Neuron-Myt1l
Neutrophil-Fpr1
NK-Klra4
T-Cd3d
Endothelium-Sox17
Epithelial-Krt23
F
Twist2
Barx1
Prelp
Msx1
Ror2
Wnt5a
G
Trem2
Gm6377
Ms4a6d
Clec4a3
Msr1
Clec4a1

Figure S1. Single-cell transcriptomic analysis of periodontal tissues identifies mesenchymal heterogeneity. (A) Single-cell RNA-seq quality control (QC) metrics. (B) scRNA-seq clustering of all cells based on UMAP projection. (C–D) UMAP visualization of major cell types in the two groups and corresponding composition plots. (E) Feature plots of representative marker genes (12 shown). Full marker lists for all 14 cell classes are provided in Supplementary Table S1. (F–G) UMAP visualization of representative mesenchymal lineage cells and Mono_Macrophage_DC marker genes.

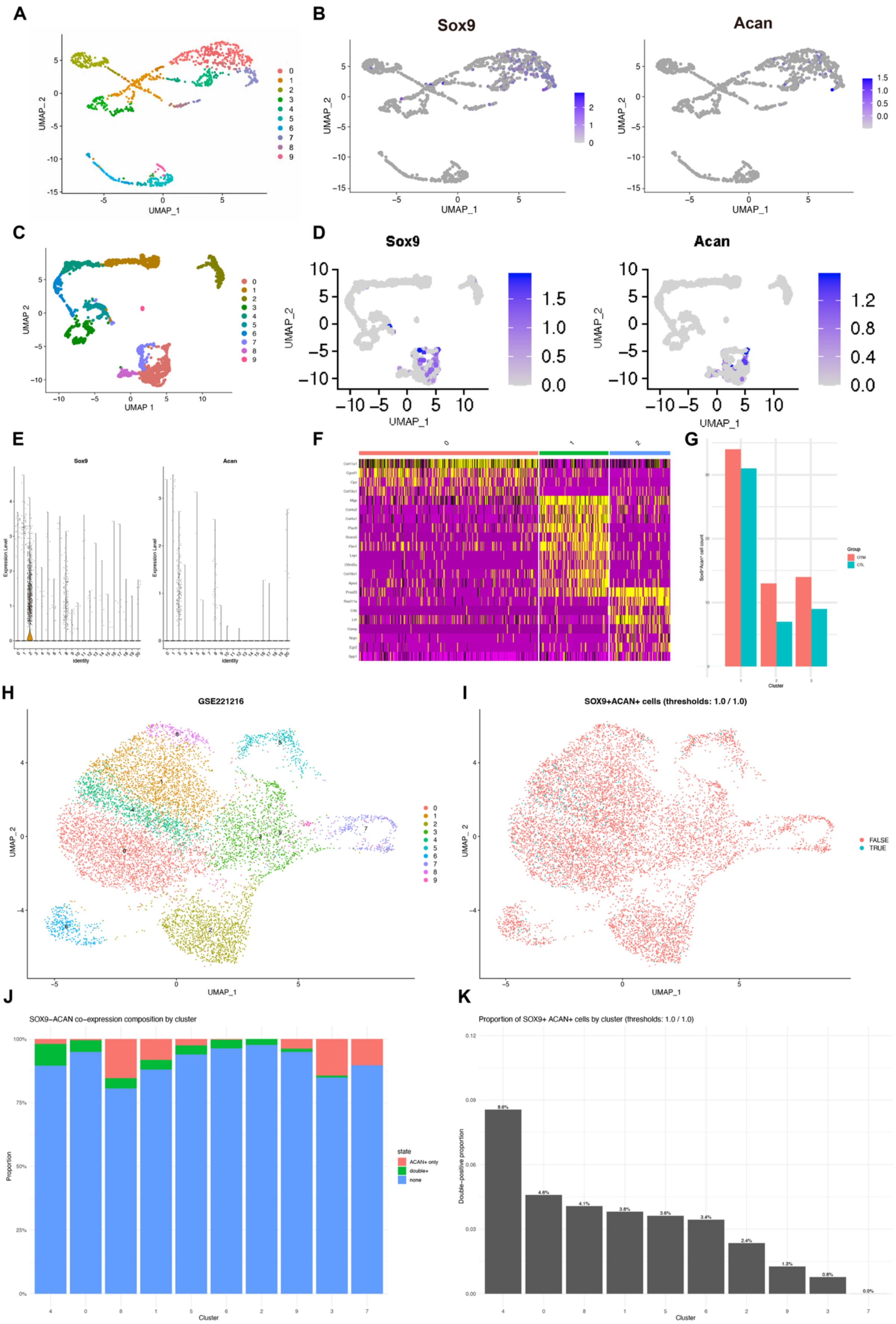
A
UMAP_1
UMAP_2
B
Sox9
Acan
UMAP_1
UMAP_2
C
UMAP 1
UMAP 2
D
Sox9
Acan
UMAP_1
UMAP_2
E
Sox9
Acan
Expression Level
Identity
F
G
Group
OTM
CTL
Cluster
H
GSE221216
UMAP_1
UMAP_2
I
SOX9+ACAN+ cells (thresholds: 1.0 / 1.0)
FALSE
TRUE
UMAP_1
UMAP_2
J
SOX9–ACAN co–expression composition by cluster
state
ACAN+ only
double+
none
Proportion
Cluster
K
Proportion of SOX9+ ACAN+ cells by cluster (thresholds: 1.0 / 1.0)
Double–positive proportion
Cluster
8.6%
4.8%
4.1%
3.8%
3.6%
3.4%
2.4%
1.3%
0.8%
0.0%

Figure S2. $Sox9^{+}Acan^{+}$ cells across multiple scRNA-seq datasets. (A–B) UMAP visualization and Sox9 or Acan FeaturePlots from the GSE160358 dataset. (C–D) UMAP with cluster annotations and corresponding FeaturePlots from the GSE 168450 dataset. (E–G) Clusterwise expression distributions of Sox9 and Acan, a marker-gene heatmap validating cluster identities, and a bar chart summarizing Sox9 or Acan positive cells from the GSE287729 dataset. (H–K) UMAP with clusters, a binary map highlighting $Sox9^{+}Acan^{+}$ cells, stacked bars showing co-expression composition (double-positive, Sox9-only, Acan-only, none), and the proportion of double-positive cells per cluster from the GSE221216 dataset. Expression values are log-normalized, double-positivity is defined as Sox9 ≥ 1.0 and Acan ≥ 1.0, and proportions reflect the fraction of $Sox9^{+}Acan^{+}$ cells within each cluster.

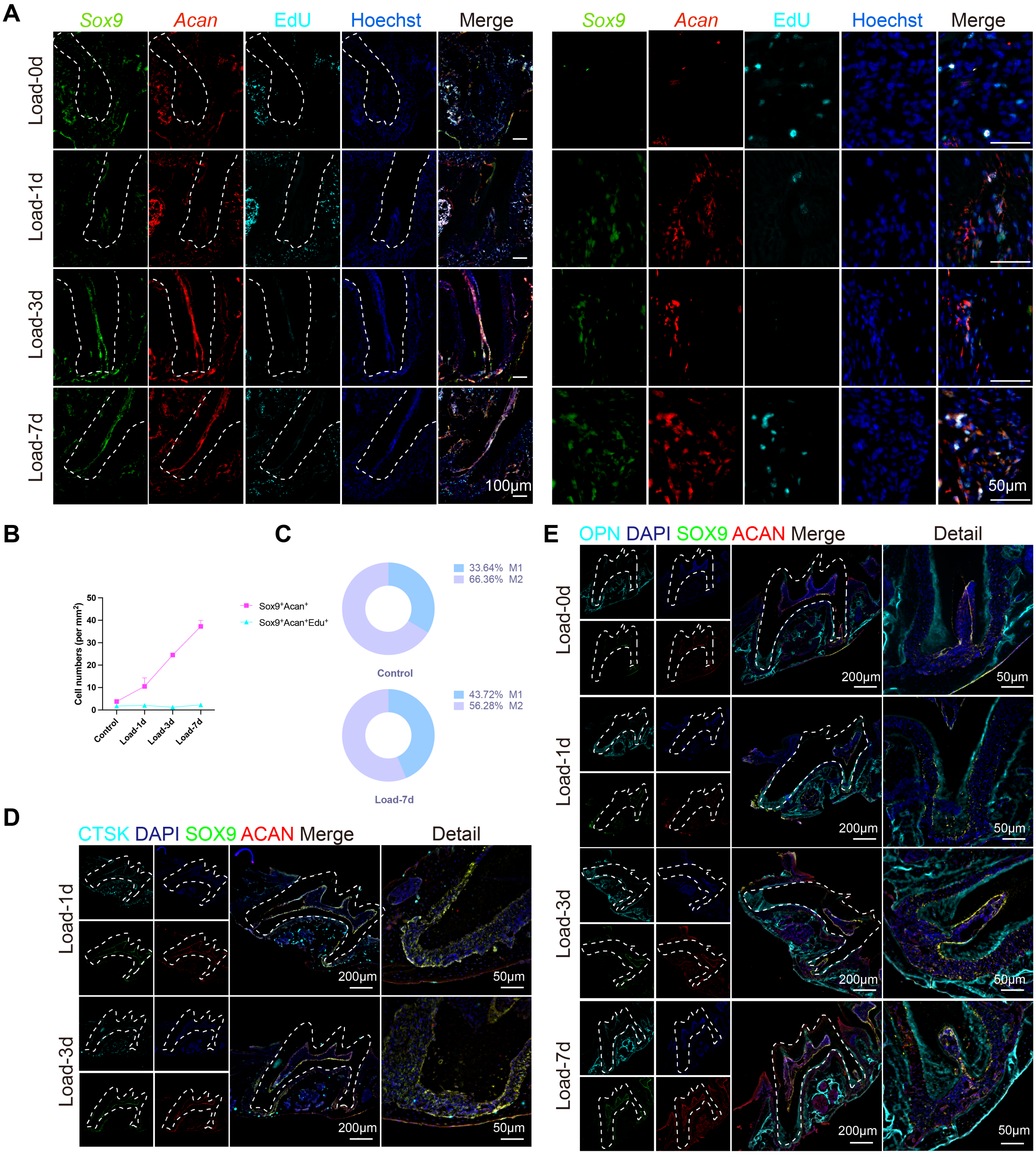

Figure S3. Sox9^{+}Acan^{+} stromal cells are enriched and activated under orthodontic force in vivo. (A) RNAscope detection of Sox9, Acan, and EdU across different loading time points, shown at low magnification (left) and high magnification (right). Single-channel and merged images are displayed as labeled in the figure. (B) Quantification of Sox9^{+}Acan^{+}Edu+ cells. Values represent mean ± SD of three independent mice data. (C) Pie charts showing proportions of CD86 (M1) and CD206 (M2) Macrophages. (D–E) mIHC of SOX9, ACAN and CTSK/OPN in periodontal ligament at different loading times. All these experiments were

performed with n = 3–6 mice per group.

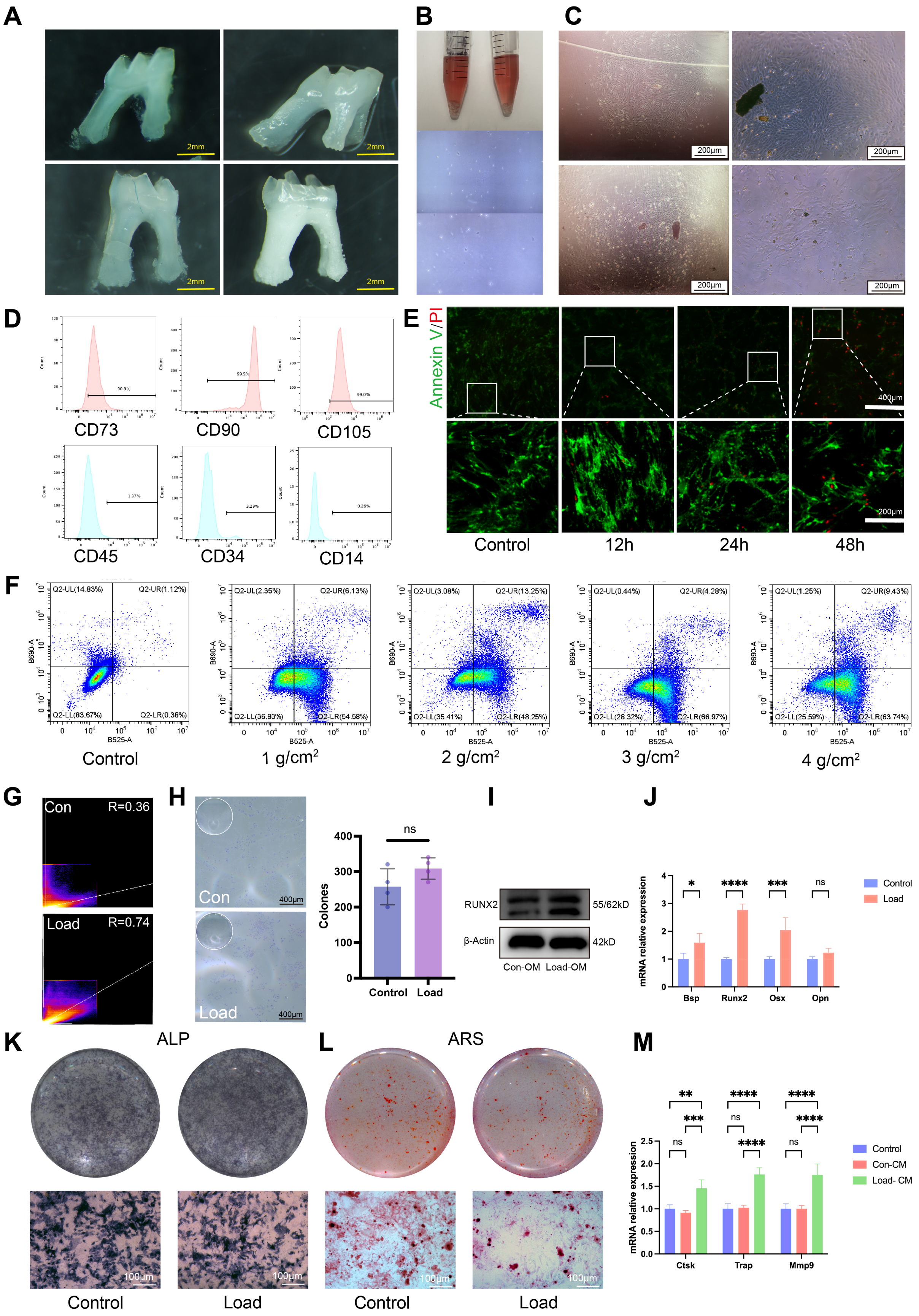
A
2mm
B
C
200μm
D
CD73
CD90
CD105
CD45
CD34
CD14
E
Annexin V/PI
400μm
200μm
Control
12h
24h
48h
F
Q2-UL(14.83%)
Q2-UR(1.12%)
Q2-LL(83.67%)
Q2-LR(0.38%)
Q2-UL(2.35%)
Q2-UR(6.13%)
Q2-LL(36.93%)
Q2-LR(54.58%)
Q2-UL(3.08%)
Q2-UR(13.25%)
Q2-LL(35.41%)
Q2-LR(48.25%)
Q2-UL(0.44%)
Q2-UR(4.28%)
Q2-LL(28.32%)
Q2-LR(66.97%)
Q2-UL(1.25%)
Q2-UR(9.43%)
Q2-LL(25.59%)
Q2-LR(63.74%)
B690-A
B525-A
Control
1 g/cm²
2 g/cm²
3 g/cm²
4 g/cm²
G
Con
R=0.36
Load
R=0.74
H
Con
Load
400μm
ns
Colones
Control
Load
I
RUNX2
55/62kD
β-Actin
42kD
Con-OM
Load-OM
J
mRNA relative expression
Control
Load
Bsp
Runx2
Osx
Opn
K
ALP
100μm
Control
Load
L
ARS
100μm
Control
Load
M
mRNA relative expression
Control
Con-CM
Load- CM
Ctsk
Trap
Mmp9

Figure S4. Isolation and characterization of periodontal stromal cells under force. (A–C) Gross morphology of dissected periodontal tissues and primary cultured cells. (D) Flow cytometry confirming MSCs surface marker expression (CD73, CD90, CD105) and absence of hematopoietic markers (CD34, CD45, CD14). (E) Annexin V/PI staining showing cell viability under force application. (F) Flow cytometry assessing apoptosis under increasing compressive forces. (G–I) Fluorescence colocalization, colony formation assay, and Western blot of RUNX2 expression in control and loaded groups. (J–M) qRT-PCR analysis, ALP and ARS staining. Data were collected from three independent experiments, (n = 3) and were analyzed by unpaired two-tailed Student's t-test (two groups) or one-way ANOVA with Tukey's post-hoc test (multiple groups). Graphs show mean ± SD, with each dot representing individual data. ns ($P \geq 0.05$), *$P < 0.05$, **$P < 0.01$, ***$P < 0.001$, ****$P < 0.0001$.

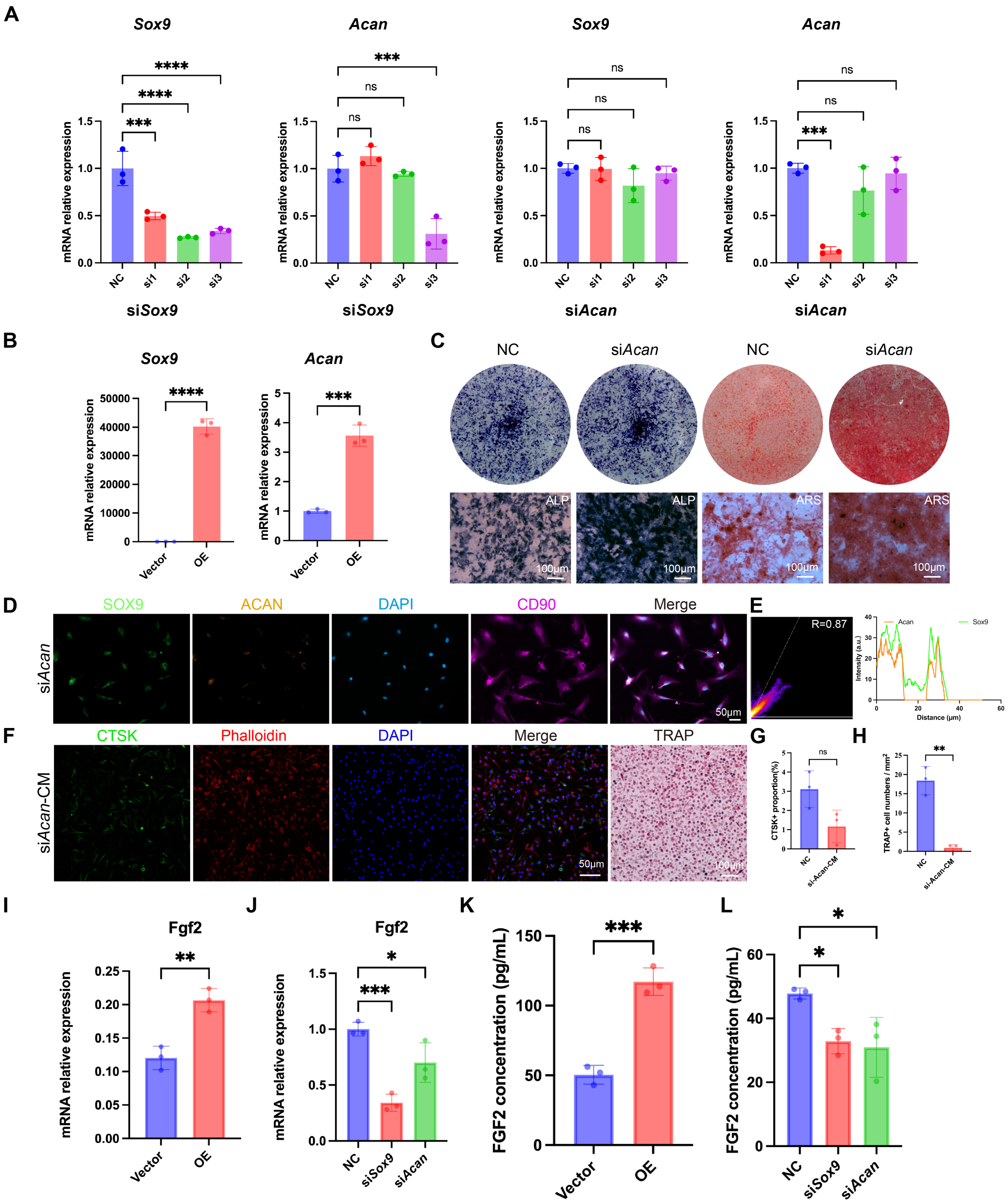

Figure S5. Functional validation of Sox9 and Acan in regulating stromal and osteoclast activity. (A–B) qPCR analysis showing the efficiency of Sox9 and Acan knockdown and overexpression. (C) ALP and ARS staining after Acan knockdown demonstrating enhanced osteogenic differentiation. (D–E) Immunofluorescence images showing colocalization of SOX9, ACAN, and CD90, with quantitative analysis of SOX9 and ACAN correlation. (F–H)

Conditioned medium experiments showing reduced CTSK expression, TRAP staining, and osteoclast numbers following Acan knockdown. (I–J) qPCR analysis of FGF2 mRNA expression after Sox9 knockdown or overexpression, and Acan knockdown. (K–L) ELISA analysis of FGF2 protein levels in culture supernatants under Sox9 knockdown or overexpression, and Acan knockdown conditions. Data were collected from three independent experiments, ($n = 3$) and were analyzed by unpaired t test. Graphs show mean ± SD, with each dot representing individual data. ns ($P \geq 0.05$), *$P < 0.05$, **$P < 0.01$, ***$P < 0.001$, ****$P < 0.0001$.

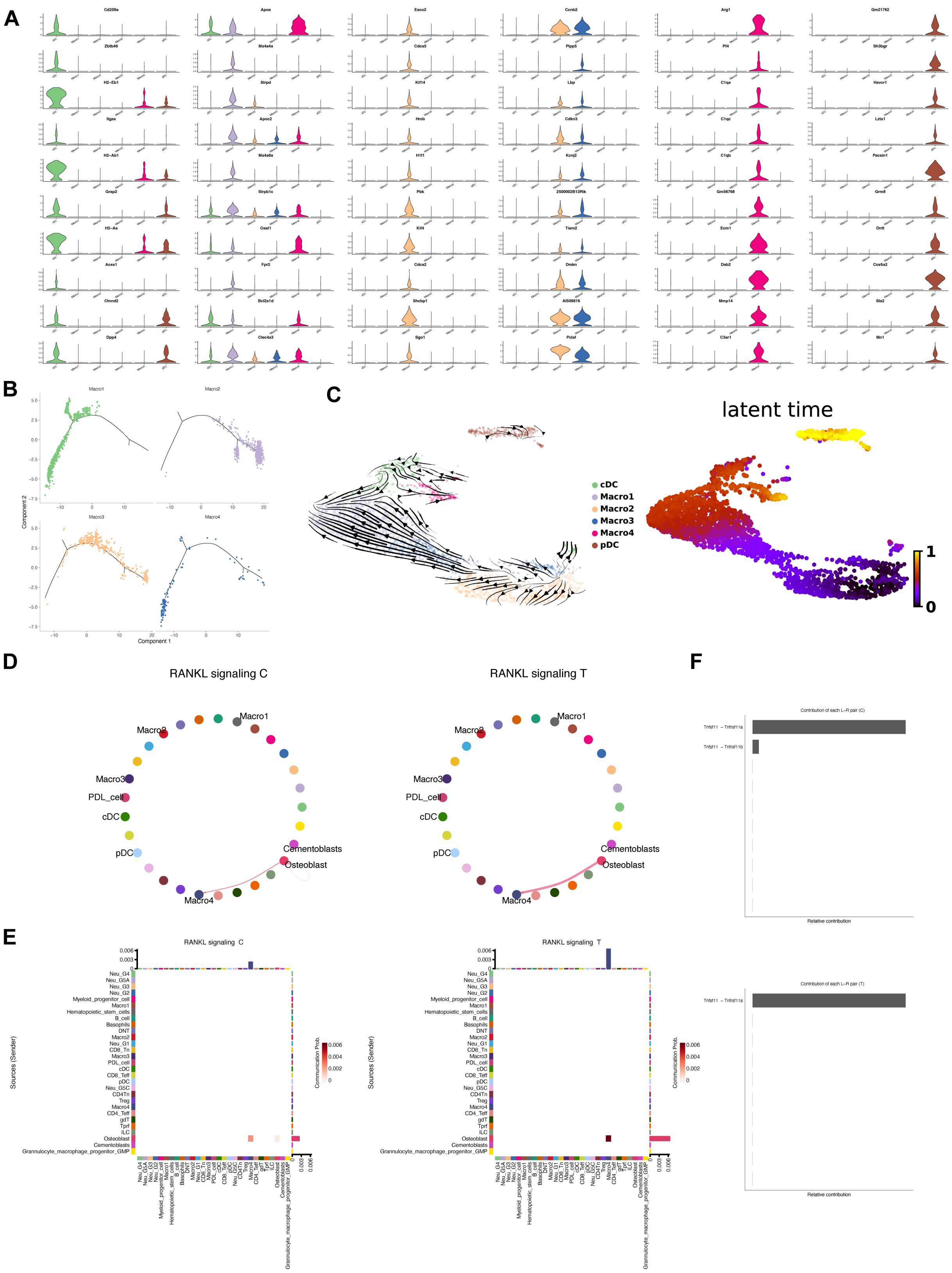

Figure S6. Characterization of macrophages and interaction analysis. (A) Violin plots showing the expression of marker genes for plasmacytoid dendritic cells (pDC), conventional dendritic

cells (cDC), and macrophages. Macrophages and dendritic cells were analyzed together as they were co-clustered within the myeloid lineage during initial classification. (B) UMAP heatmap illustrating the distribution and transcriptional profiles of macrophages. (C) Pseudotime trajectory, RNA velocity, and latent time analyses revealing the dynamic transition of macrophage states. (D–F) Alterations in RANKL–RANK interactions between osteoblasts and Macro4 across C and T groups.

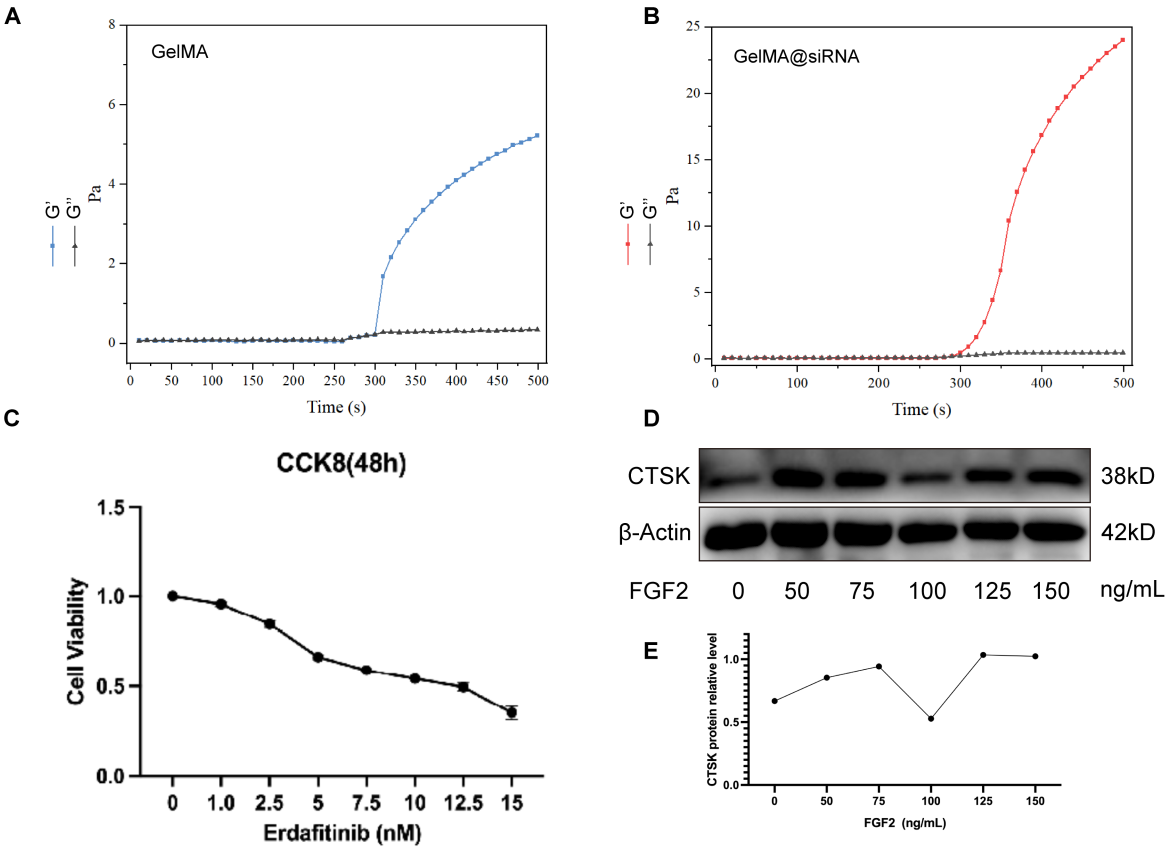

Figure S7. (A–B) Rheological properties of GelMA (A) and GelMA@siRNA (B) hydrogels, showing storage modulus (G′) and loss modulus (G″) as a function of time during gelation. (C) Cytotoxicity of the FGFR inhibitor Erdafitinib at increasing concentrations (0–15 nM) assessed by CCK-8 assay after 48 h culture. (D, E) Western blot analysis (D) and quantification (E) of CTSK protein expression in bone marrow–derived macrophages treated with recombinant FGF2 at the indicated concentrations (0–150 ng/mL). β-Actin served as loading control.

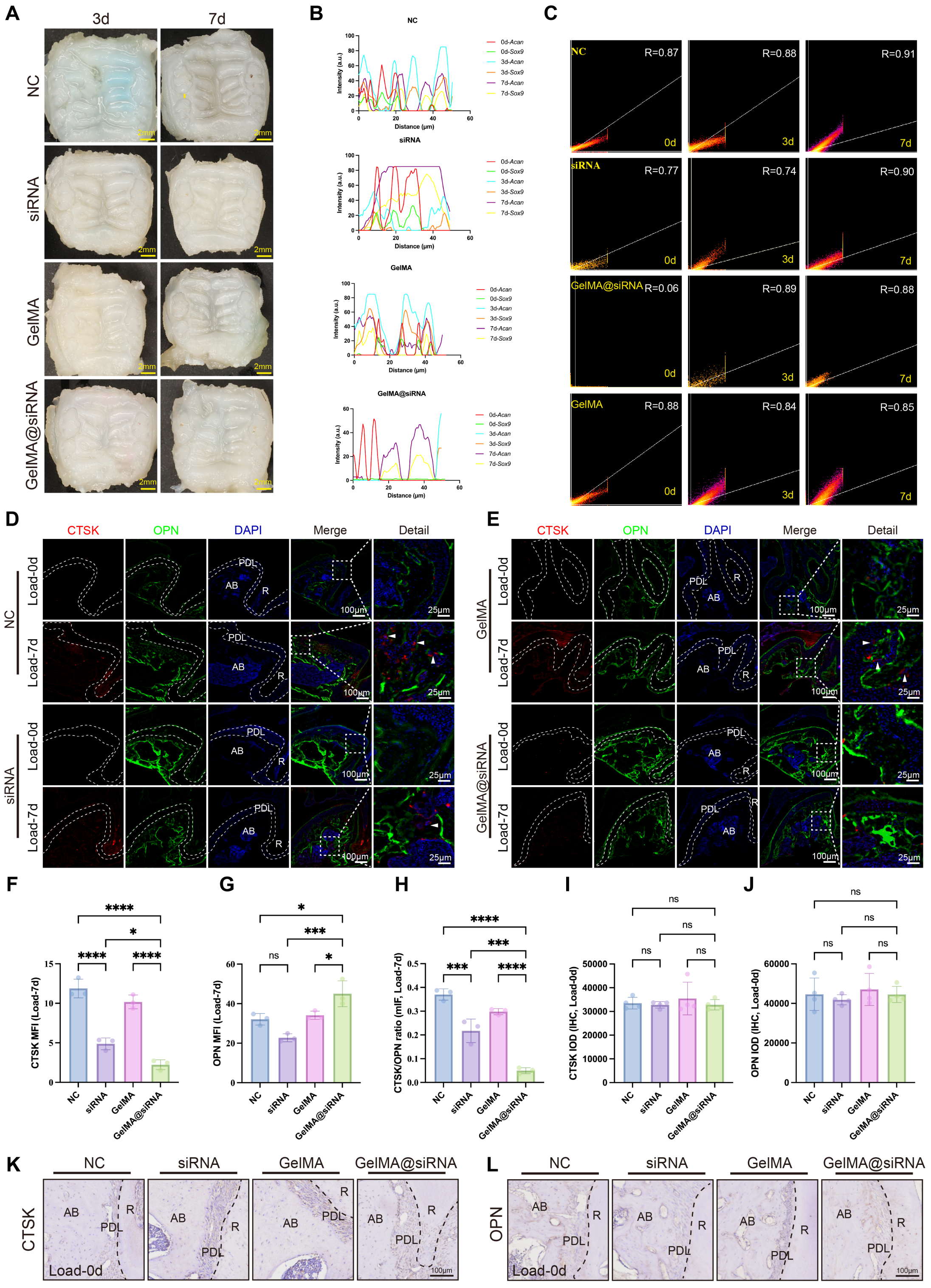

Figure S8. Tissue-Level Effects of Local Sox9 Silencing. (A) Representative gross images of the mouse maxilla in the tooth-movement model, showing the right side as the loading group. (B–C) RNAscope-based *Sox9–Acan* colocalization analysis after force loading in the NC, siRNA, GelMA, and GelMA@siRNA groups. (D–E) mIF images showing CTSK and OPN

expression at Load-0d and Load-7d across the NC, siRNA (D), GelMA, and GelMA@siRNA (E) groups, each presented at both high and low magnification with PDL, root, and alveolar bone (AB) regions indicated, with white arrows marking representative regions used for cross-group visual comparison. (F–H) Quantification of CTSK and OPN mean fluorescence intensity (MFI) and the CTSK/OPN ratio at Load-7d across the NC, siRNA, GelMA, and GelMA@siRNA groups (n = 3–6 per group). (I–L) Immunohistochemical staining of CTSK and OPN at Load-0d in the NC, siRNA, GelMA, and GelMA@siRNA groups, with corresponding IOD quantification showing no significant differences. Graphs show mean ± SD, with each dot representing individual data. ns ($P \geq 0.05$), *$P < 0.05$, **$P < 0.01$, ***$P < 0.001$, ****$P < 0.0001$.

Supplementary Tables

Table S1. Marker genes for major cell types and subclusters.

Cell type	Key markers
B_cell	Ebf1, Cd79a, Blnk
Basophils	Cd200r3, Gata2, Tec
Mesenchymal lineage cell	Col1a1, Col1a2, Bgn, Igfbp7
Endothelium	Emcn, Esam, Cdh5, Ptprb
Epithelial	Krt5, Krt14, Trp63
Grannulocyte_macrophage_progenitor_GMP	Ms4a3,Cebpe, Mki67
Hematopoietic_stem_cells	Adgrg1, Msi2, Etv6
Mono_macrophage_DC	Mpeg1, Ccr2, Ctss, Ifi30, Npc2, Psap
Myeloid_progenitor_cell	Ms4a3, Mpo, Prtn3, Elane
Neuron	Snap25, Syt1, Rims2
Neutrophil	S100a8, S100a9, Lcn
NK cell	Eomes, Il2rb, Gzma, Ccl5

Schwann cell	Mpz,Cdh19, Plp1
T cell	Itk, Skap1, Grap2, Prkch, Camk4

Subclusters	Key markers
Fibroblast	Mfap5, Dpt, Col14a1, Dcn, Fbln2,
Osteoblast	Alpl, Bglap2, Postn
Cementoblast	Dmp1, Ibsp, Mfge8
Mesenchymal stem cell	Msx2, Fgfr1, Slc20a2, Limch1, Rarb, Pclo
Sox9+Acan+	Sox9, Acan

Table S2. Antibodies used for western blotting, immunofluorescence staining, flow cytometry, and immunohistochemistry staining.

Antibody	Vendor	Catalog No.	Host & Isotype	Recommended Dilution
SOX9 [EPR14335-78]	Abcam	ab185966	Rabbit mAb	WB 1:1000; IHC/IF 1:200
Aggrecan	Affinitiy	DF7561	Rabbit pAb	WB 1:1000; IHC 1:200
FGF1	Abclonal	A0685	Rabbit mAb	WB 1:1000
FGF2	Abclonal	A11488	Rabbit mAb	WB 1:1000
FGFR1	Affinity	AF6156	Rabbit pAb	WB 1:1000
FGFR2	Affinity	AF0159	Rabbit pAb	WB 1:1000
p44/42 MAPK (Erk1/2)	CST	9102	Rabbit pAb	WB 1:1000
Phospho-p44/42	CST	9101 / 4370	Rabbit pAb	WB 1:1000

MAPK (Thr202/Tyr204)				
SAPK/JNK	CST	9252	Rabbit pAb	WB 1:1000
Phospho-SAPK/JNK (Thr183/Tyr185)	CST	9251 / 9255	Rabbit pAb	WB 1:1000
p38 MAPK	CST	9212	Rabbit pAb	WB 1:1000
Phospho-p38 MAPK (Thr180/Tyr182)	CST	9211 / 4511	Rabbit pAb	WB 1:1000
Akt (pan)	CST	9272	Rabbit pAb	WB 1:1000
Phospho-Akt (Ser473)	CST	4060 / 9271	Rabbit mAb XP® (D9E)	WB 1:1000
FAK (D2R2E)	CST	13009	Rabbit mAb	WB 1:1000
Phospho-FAK (Tyr397)	CST	3283	Rabbit mAb	WB 1:1000
RUNX2 (D1L7F)	CST	12556	Rabbit mAb	WB 1:1000
Cathepsin K	CST	57056	Rabbit mAb	WB 1:1000; IHC 1:200
Osteopontin	Servicebio	GB120018-100	Rabbit mAb	WB 1:1000; IHC/IF 1:200
β-Actin (13E5)	CST	4970	Rabbit mAb	WB 1:2000; IHC/IF 1:200
anti-mouse CD90	Elabscience	E-AB-F1094E	Mouse IgG1 Isotype Control	Flow 1:20–1:100

anti-mouse CD73	Elabscience	E-AB-F1089E	Mouse IgG1 Isotype Control	Flow 1:20–1:100
anti-mouse CD105	Elabscience	E-AB-F1233E	Mouse IgG1 Isotype Control	Flow 1:20–1:100
anti-mouse CD45	Elabscience	E-AB-F1136E	Mouse IgG1 Isotype Control	Flow 1:20–1:100
anti-mouse CD34	Elabscience	E-AB-F1284E	Mouse IgG1 Isotype Control	Flow 1:20–1:100
anti-mouse CD14	Elabscience	E-AB-F1176E	Mouse IgG1 Isotype Control	Flow 1:20–1:100
Mouse IgG1	Elabscience	E-AB-F09792E		Use matched to test Ab

Table S3. Sequences of primers used in qRT-PCR (synthesized by Tsingke Biotechnology, Beijing, China).

Gene Symbol	Species	Primer Sequence
Runx2	mouse	F: CCTGAACTCTGCACCAAGTCCT
		R: TCATCTGGCTCAGATAGGAGGG
Bsp	mouse	F: AATGGAGACGGCGATAGTTCCG
		R: GGAAAGTGTGGAGTTCTCTGCC
Osx	mouse	F: GGCTTTTCTGCGGCAAGAGGTT
		R: CGCTGATGTTTGCTCAAGTGGTC
Opn	mouse	F: GCTTGGCTTATGGACTGAGGTC

		R: CCTTAGACTCACCGCTCTTCATG
Ctsk	mouse	F: AGCAGAACGGAGGCATTGACTC
		R: CCCTCTGCATTTAGCTGCCTTTG
Trap	mouse	F: GCGACCATTGTTAGCCACATACG
		R: CGTTGATGTCGCACAGAGGGAT
Mmp9	mouse	F: GCTGACTACGATAAGGACGGCA
		R: TAGTGGTGCAGGCAGAGTAGGA
Sox9	mouse	F: CACACGTCAAGCGACCCATGAA
		R: TCTTCTCGCTCTCGTTCAGCAG
Acan	mouse	F: CAGGCTATGAGCAGTGTGATGC
		R: GCTGCTGTCTTTGTCACCCACA
β-actin	mouse	F: CATTGCTGACAGGATGCAGAAGG
		R: TGCTGGAAGGTGGACAGTGAGG

Table S4. siRNA information (synthesized by Tsingke Biotechnology, Beijing, China).

Name	Sequence(5'-3')	Base	Purification	MW	Modification	OD/Tube	ODs
siSOX9-1	CGACGUGGACAUCGGUGAA(dT)(dT)	21	HPLC	6748.19	/	0.50	2.00
siSOX9-1	UUCACCGAUGUCCACGUCG(dT)(dT)	21	HPLC	6582.02			2.00
siSOX	CCACCUUCACUUACAUG	21	HPLC	6534.			2.0

9-2	AA(dT)(dT)			02			0
siSOX9-2	UUCAUGUAAGUGAAGGUGG(dT)(dT)	21	HPLC	6751.16			2.00
siSOX9-3	CAAAGUUGAUCUGAAGCGA(dT)(dT)	21	HPLC	6717.18			2.00
siSOX9-3	UCGCUUCAGAUCAACUUUG(dT)(dT)	21	HPLC	6568.00			2.00
siACAN-1	GAAUGACAGGACUAUCGAA(dT)(dT)	21	HPLC	6740.22	/	0.50	2.00
siACAN-1	UUCGAUAGUCCUGUCAUUC(dT)(dT)	21	HPLC	6544.96			2.00
siACAN-2	GGACAGUCCUCUGGUAUAA(dT)(dT)	21	HPLC	6670.11			2.00
siACAN-2	UUAUACCAGAGGACUGUCC(dT)(dT)	21	HPLC	6630.08			2.00
siACAN-3	CACUCAGCUUCCUACUGAA(dT)(dT)	21	HPLC	6550.02			2.00
siACAN-3	UUCAGUAGGAAGCUGAGUG(dT)(dT)	21	HPLC	6750.17			2.00

Table S5. Modification strategy for in vivo application of si SOX9-3 (synthesized by Tsingke Biotechnology, Beijing, China).

sense	CAAA(mG)(mU)(mU)(mG)A(mU)C(mU)(mG)AA(mG)C(mG)A(dT)(dT)	21	HPLC	14799.35	(mG)'5,(mU)'4,(dT)'2,3'Cholesteryl	1.00	4.00

anti sens e	(mU)C(mG)C(mU)(mU)CA(mG)A(m U)CAAC(mU)(mU)(mU)(mG)(dT)(d T)	2 1	H PL C	147 99.3 5	(mG)'3,(mU)'7,(dT)'2,3'Cholester yl	1. 0 0	4. 0 0

Table S6. *Sox9* Overexpression Plasmid Information (synthesized by Tsingke Biotechnology, Beijing, China).

Item	Details
Gene	Sox9 (mouse)
Vector backbone	pcDNA3.1(+) (Ampicillin resistance, ID: ZT000103)
Insert size	1,536 bp
Cloning sites	BamHI (5′) – EcoRI (3′)
Sequence source	Synthetic (codon-preserved, no extra sequence introduced)
Plasmid grade	EndoFree (< 0.1 EU/µg)
Deliverables	1 tube 4 µg lyophilized plasmid; 1 tube Stablized E. coli Top10; 1 tube 100 µg liquid plasmid
Work cycle	15–21 calendar days (synthesis + plasmid preparation)
Service provider	Tsingke Biotechnology Co., Ltd. (Beijing, China)
Sequence	Full-length cDNA corresponding to mouse Sox9 (GenBank accession cross-verified; cloning sequence provided upon request)

The Sox9 coding sequence (1,536 bp) was synthesized and cloned into pcDNA3.1(+) between BamHI and EcoRI restriction sites. Plasmids were prepared using EndoFree purification (< 0.1 EU/µg), suitable for mammalian cell transfection. Both lyophilized plasmid DNA and E. coli Stab-Top10 glycerol stocks were provided to ensure long-term storage and

reproducibility.

Supplementary Methods

Image Analysis

Software environment

QuPath: v0.4.3 (University of Edinburgh, UK)

Python: 3.9.18

Packages: numpy 1.23.5, pandas 1.5.3, scipy 1.10.1, matplotlib 3.7.1, seaborn 0.12.2

Step 1. Cell detection in QuPath

Whole-slide immunofluorescence images were opened in QuPath (v0.4.3).

Regions of interest (ROIs) were manually annotated around the periodontal ligament (PDL) area adjacent to the root surface.

The “Cell detection” command was applied with the following parameters:

Requested pixel size: 0.5 µm

Background radius: 8 µm

Median filter radius: 0 µm

Sigma: 1.5 µm

Minimum cell area: 25 μm^2

Maximum cell area: 400 μm^2

Positive cells were classified based on fluorescence thresholds of nuclear/cytoplasmic

channels:

Sox9$^+$ cells: nuclear SOX9 signal > threshold T1

CD206$^+$ cells: cytoplasmic CD206 signal > threshold T2

CD86$^+$ cells: cytoplasmic CD86 signal > threshold T3

Positivity thresholds (T_1 for SOX9, T_2 for CD206, T_3 for CD86) were computed per image using Otsu's method within ROI masks, and applied to classify positive cells. All images were acquired under identical settings without intensity rescaling.

Cell coordinates (centroid X, Y) and classification (Sox9, CD206, CD86, negative) were exported as .csv using the QuPath "Export measurements" function.

Step 2. Spatial analysis in Python

Distances between Sox9$^+$ cells and CD206$^+$/CD86$^+$ cells were calculated using Euclidean distance. For each Sox9$^+$ cell, all neighboring CD206$^+$ or CD86$^+$ cells were assigned to bins: 0–10 µm, 10–20 µm, 20–30 µm, 30–40 µm, and >40 µm.

Python code used:

```
import pandas as pd

import numpy as np

from scipy.spatial import distance

# Load QuPath exported data

data = pd.read_csv("QuPath_cell_export.csv")

# Split by cell type

sox9_cells = data[data['Class'] == 'Sox9'][['Centroid X µm','Centroid Y µm']]
```

```
cd206_cells = data[data['Class'] == 'CD206'][['Centroid X µm','Centroid Y µm']]

cd86_cells    = data[data['Class'] == 'CD86'][['Centroid X µm','Centroid Y µm']]

# Define distance bins

bins = [0,10,20,30,40,1e6]

labels = ['0-10','10-20','20-30','30-40','>40']

def count_neighbors(sox9_df, target_df, bins, labels):

    results = []

    for i, sox9 in sox9_df.iterrows():

        dists = distance.cdist([sox9.values], target_df.values, 'euclidean')[0]

        hist, _ = np.histogram(dists, bins=bins)

        results.append(hist)

    results = np.array(results)

    return pd.DataFrame(results, columns=labels)

# Count CD206 neighbors

cd206_counts = count_neighbors(sox9_cells, cd206_cells, bins, labels)

# Count CD86 neighbors

cd86_counts = count_neighbors(sox9_cells, cd86_cells, bins, labels)

# Aggregate (mean ± SD per Sox9+ cell)

summary_cd206 = cd206_counts.describe().loc[['mean','std']]
```

```
summary_cd86    = cd86_counts.describe().loc[['mean','std']]

# Save results

summary_cd206.to_csv("cd206_neighbor_summary.csv")

summary_cd86.to_csv("cd86_neighbor_summary.csv")
```

Step 3. Output

Results were summarized as the average number (± SD) of CD206^{+} or CD86^{+} cells within each distance bin around Sox9^{+} cells. Visualization was performed using matplotlib/seaborn to generate bar plots for each distance range.
